# Supplementary figures and images for: An NMR sample preparation case study: Considerations for the self-destructive protease caspase-6
Source: PLoS One. 2025 Nov 21;20(11):e0337291. doi: 10.1371/journal.pone.0337291 (PMC12637907; doi:10.1371/journal.pone.0337291)

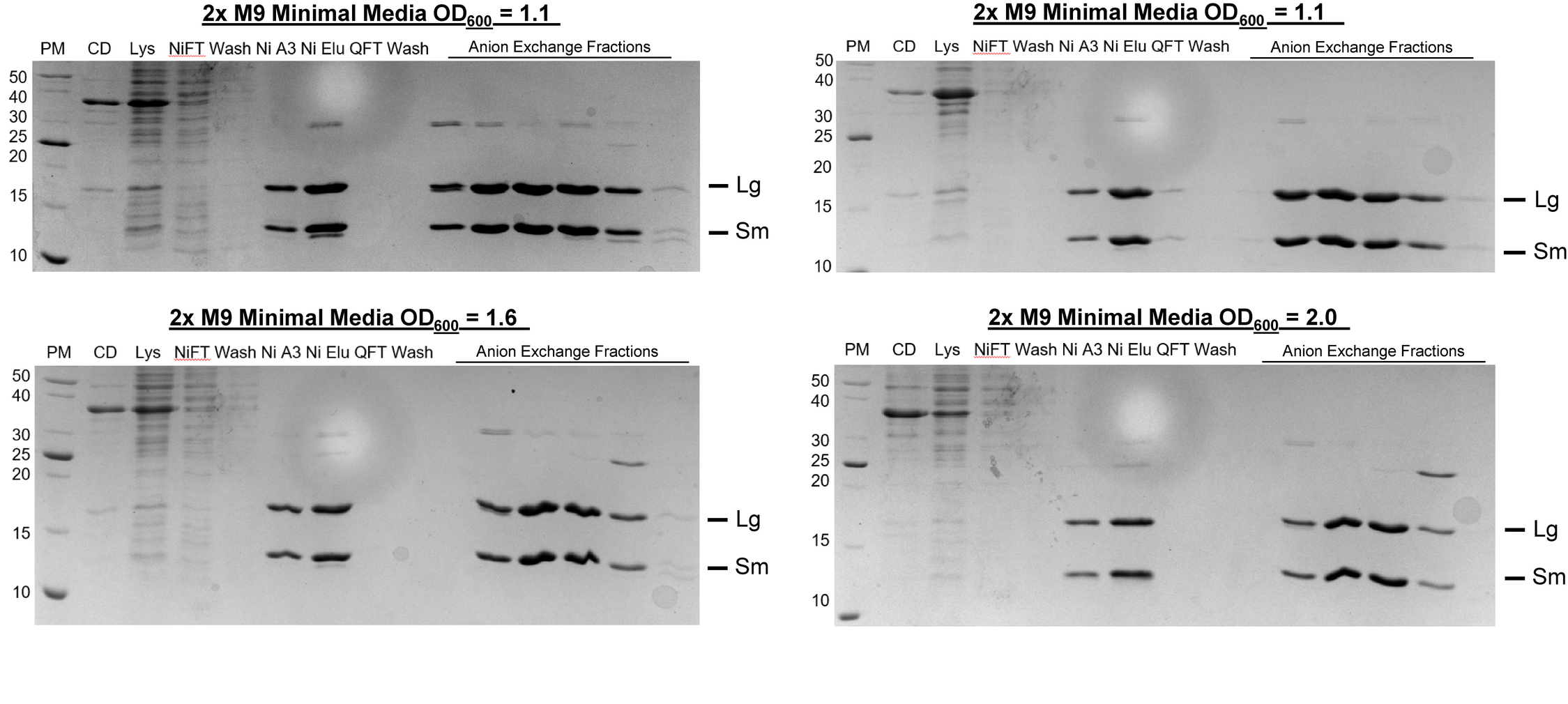

Supplement: S1 Fig — Increasing OD600 did not appear to increase remaining by-products in the final purified casp-6. Fractions from the purification include cell debris after cell lysis (CD), cellular lysate (Lys), Ni column flow-through (NiFT), Ni column wash, Ni column elution (Ni Elu), anion-exchange flow-through (QFT), anion-exchange wash (Q Wash), and individual fractions from anion-exchange. (TIF) [file pone.0337291.s001.tif]

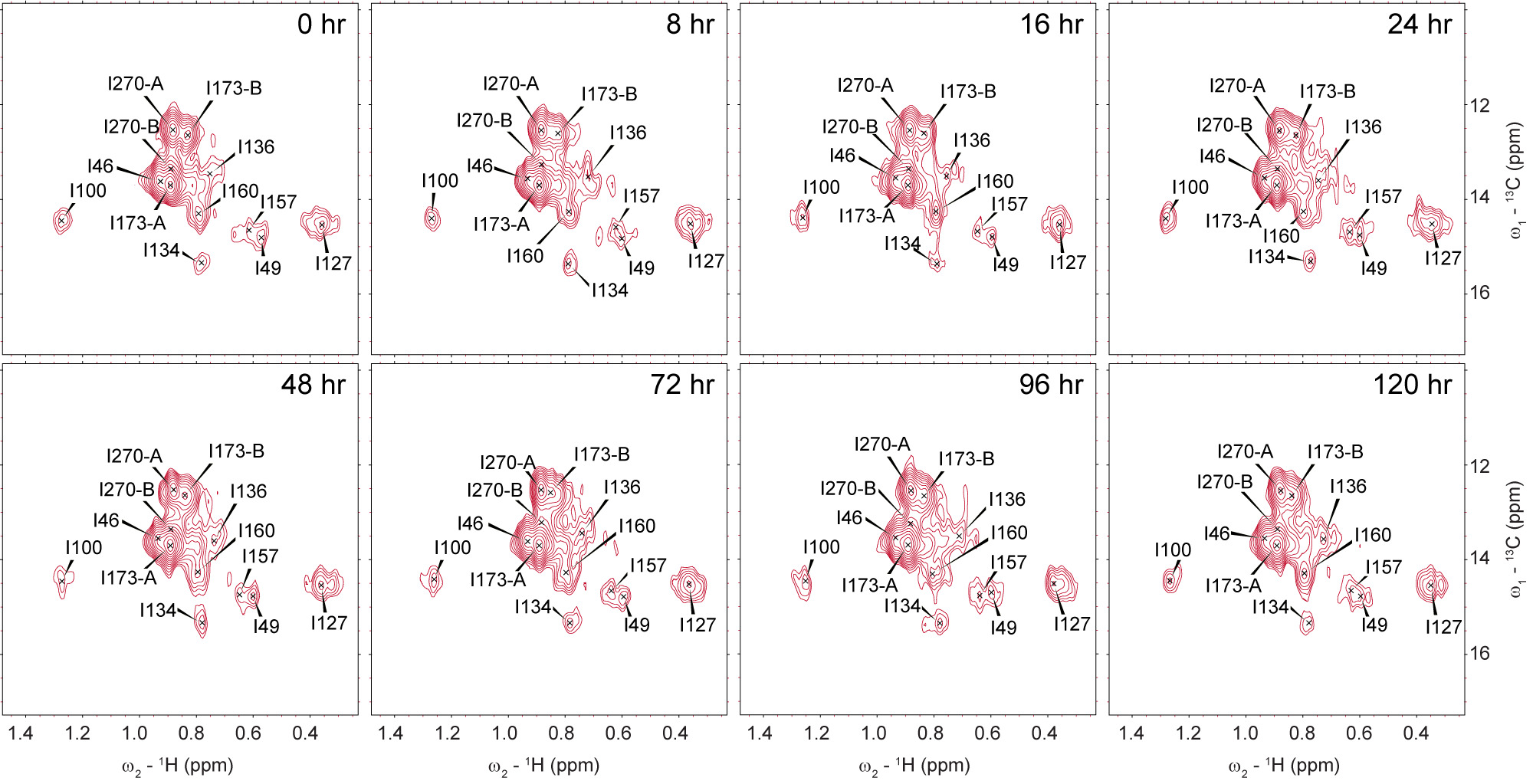

Supplement: S2 Fig — A series of 1H-13C HMQC spectra for Ileδ1-13CH3-labeled casp-6 D179 CT at 25°C and 600 MHz show no significant loss in signal intensity for up to 120 hours, suggesting a stable and soluble sample throughout this period. (TIF) [file pone.0337291.s002.tif]

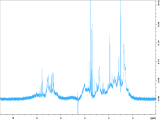

Supplement: S1 Data — Data are categorized by its respective main text figure. Files are titled with a description of contents. Exported text instructions and images for optimized purification protocols are in the Purification Methods folder. (ZIP) [file pone.0337291.s007.zip › S1_Data/Fig 6 - 1H NMR Files/C6 D179 CT in Phosphate/10/pdata/1/thumb.png]

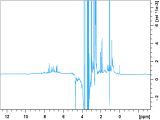

Supplement: S1 Data — Data are categorized by its respective main text figure. Files are titled with a description of contents. Exported text instructions and images for optimized purification protocols are in the Purification Methods folder. (ZIP) [file pone.0337291.s007.zip › S1_Data/Fig 6 - 1H NMR Files/C6 D179 CT NaCl Testing/1/pdata/1/thumb.png]

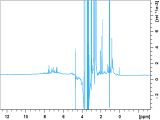

Supplement: S1 Data — Data are categorized by its respective main text figure. Files are titled with a description of contents. Exported text instructions and images for optimized purification protocols are in the Purification Methods folder. (ZIP) [file pone.0337291.s007.zip › S1_Data/Fig 6 - 1H NMR Files/C6 D179 CT NaCl Testing/2/pdata/1/thumb.png]

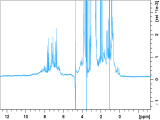

Supplement: S1 Data — Data are categorized by its respective main text figure. Files are titled with a description of contents. Exported text instructions and images for optimized purification protocols are in the Purification Methods folder. (ZIP) [file pone.0337291.s007.zip › S1_Data/Fig 6 - 1H NMR Files/C6 D179 CT NaCl Testing/3/pdata/1/thumb.png]

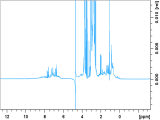

Supplement: S1 Data — Data are categorized by its respective main text figure. Files are titled with a description of contents. Exported text instructions and images for optimized purification protocols are in the Purification Methods folder. (ZIP) [file pone.0337291.s007.zip › S1_Data/Fig 6 - 1H NMR Files/C6 D179 CT NaCl Testing/4/pdata/1/thumb.png]

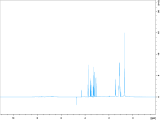

Supplement: S1 Data — Data are categorized by its respective main text figure. Files are titled with a description of contents. Exported text instructions and images for optimized purification protocols are in the Purification Methods folder. (ZIP) [file pone.0337291.s007.zip › S1_Data/Fig 6 - 1H NMR Files/Casp6_1D_BOG_Optimization/3/pdata/1/thumb.png]
