# Supplementary material for: An NMR sample preparation case study: Considerations for the self-destructive protease caspase-6
Source: PLoS One. 2025 Nov 21;20(11):e0337291. doi: 10.1371/journal.pone.0337291 (PMC12637907; doi:10.1371/journal.pone.0337291)
Supplement: S1 Table — Minimal media as outlined here, is carefully composed of specific salts, minerals, vitamins, a single carbon source, and a single nitrogen source, whereas the complex ingredients in 2xYT provide an array of nutrients. (DOCX) [file pone.0337291.s003.docx]

| **2x M9 Minimal Media** | |
| --- | --- |
| **Ingredient** | **Concentration** |
| Na_2_HPO_4_ | 13.56 g/L |
| KH_2_PO_4_ | 6 g/L |
| NaCl | 1 g/L |
| Glucose | 2 g/L |
| NH_4_Cl | 1 g/L |
| Ampicillin | 100 mg/L |
| Thiamine | 10 µg/mL |
| Nicotinic acid | 10 µg/mL |
| Calcium pantothenate | 10 µg/mL |
| Biotin | 10 µg/mL |
| MgSO_4_ | 2 mM |
| CaCl_2_ | 150 µM |
| Na_2_SO_4_ | 15 mM |
| FeCl_3_ | 200 µM |
|  |  |
| **2xYT** | |
| **Ingredient** | **Concentration** |
| Yeast Extract | 10 g/L |
| Tryptone | 16 g/L |
| NaCl | 5 g/L |
| Ampicillin | 100 mg/L |
