# Supplementary material for: An NMR sample preparation case study: Considerations for the self-destructive protease caspase-6
Source: PLoS One. 2025 Nov 21;20(11):e0337291. doi: 10.1371/journal.pone.0337291 (PMC12637907; doi:10.1371/journal.pone.0337291)
Supplement: S2 Table — Buffer combinations used to assess casp-6 activity by cleavage of VEID-amc fluorogenic substrate. (DOCX) [file pone.0337291.s004.docx]

| **Buffering Agent** | **NaCl Concentration** | **Additives** |
| --- | --- | --- |
| 100 mM Phosphate pH 7.4 | 120 mM | 5 mM DTT |
| 100 mM Phosphate pH 7.4 | 120 mM | 5 mM DTT, 1 mM EDTA |
| 100 mM Phosphate pH 7.4 | 120 mM | 5 mM DTT, 0.1% w/v octyl-glucoside |
| 100 mM Phosphate pH 7.4 | 120 mM | 5 mM DTT, 500 mM Na_2_SO_4_ |
| 100 mM Phosphate pH 7.4 | 120 mM | 5 mM DTT, 1 mM EDTA, 500 mM Na_2_SO_4_ |
| 100 mM Phosphate pH 7.4 | 120 mM | 5 mM DTT, 0.1% w/v octyl-glucoside, 500 mM Na_2_SO_4_ |
| 100 mM Phosphate pH 7.4 | 120 mM | 5 mM DTT, 1 mM EDTA, 0.1% w/v octyl-glucoside |
| 100 mM Phosphate pH 7.4 | 120 mM | 5 mM DTT, 1 mM EDTA, 0.1% w/v octyl-glucoside, 500 mM Na_2_SO_4_ |
| 50 mM Tris pH 7.5 | 120 mM | 5 mM DTT |
| 50 mM Tris pH 7.5 | 120 mM | 5 mM DTT, 1 mM EDTA |
| 50 mM Tris pH 7.5 | 120 mM | 5 mM DTT, 0.1% w/v octyl-glucoside |
| 50 mM Tris pH 7.5 | 120 mM | 5 mM DTT, 500 mM Na_2_SO_4_ |
| 50 mM Tris pH 7.5 | 120 mM | 5 mM DTT, 1 mM EDTA, 500 mM Na_2_SO_4_ |
| 50 mM Tris pH 7.5 | 120 mM | 5 mM DTT, 0.1% w/v octyl-glucoside, 500 mM Na_2_SO_4_ |
| 50 mM Tris pH 7.5 | 120 mM | 5 mM DTT, 1 mM EDTA, 0.1% w/v octyl-glucoside |
| 50 mM Tris pH 7.5 | 120 mM | 5 mM DTT, 1 mM EDTA, 0.1% w/v octyl-glucoside, 500 mM Na_2_SO_4_ |
| 50 mM HEPES pH 7.5 | 120 mM | 5 mM DTT |
| 50 mM HEPES pH 7.5 | 120 mM | 5 mM DTT, 1 mM EDTA |
| 50 mM HEPES pH 7.5 | 120 mM | 5 mM DTT, 0.1% w/v octyl-glucoside |
| 50 mM HEPES pH 7.5 | 120 mM | 5 mM DTT, 500 mM Na_2_SO_4_ |
| 50 mM HEPES pH 7.5 | 120 mM | 5 mM DTT, 1 mM EDTA, 500 mM Na_2_SO_4_ |
| 50 mM HEPES pH 7.5 | 120 mM | 5 mM DTT, 0.1% w/v octyl-glucoside, 500 mM Na_2_SO_4_ |
| 50 mM HEPES pH 7.5 | 120 mM | 5 mM DTT, 1 mM EDTA, 0.1% w/v octyl-glucoside |
| 50 mM HEPES pH 7.5 | 120 mM | 5 mM DTT, 1 mM EDTA, 0.1% w/v octyl-glucoside, 500 mM Na_2_SO_4_ |
| 50 mM Tris pH 7.5 | 60 mM | 5 mM DTT |
| 50 mM Tris pH 7.5 | 120 mM | 5 mM DTT |
| 50 mM Tris pH 7.5 | 400 mM | 5 mM DTT |
| 100 mM Na_3_PO_4_ pH 7.4 | 60 mM | 5 mM DTT |
| 100 mM Na_3_PO_4_ pH 7.4 | 120 mM | 5 mM DTT |
| 100 mM Na_3_PO_4_ pH 7.4 | 400 mM | 5 mM DTT |
| 100 mM NaOAc pH 4.6 | 60 mM | 5 mM DTT |
| 100 mM NaOAc pH 4.6 | 120 mM | 5 mM DTT |
| 100 mM NaOAc pH 4.6 | 400 mM | 5 mM DTT |
