# Supplementary material for: An NMR sample preparation case study: Considerations for the self-destructive protease caspase-6
Source: PLoS One. 2025 Nov 21;20(11):e0337291. doi: 10.1371/journal.pone.0337291 (PMC12637907; doi:10.1371/journal.pone.0337291)
Supplement: S3 Table — A tabular presentation of the sample conditions, nucleus/experiment, solvent, solvent suppression, reference, and temperature information for the presented NMR spectra. (DOCX) [file pone.0337291.s005.docx]

| **Spectrum** | **Sample** | **Nucleus/**  **Experiment** | **Solvent** | **Solvent Suppression** | **Referencing** | **Temperature (K)** |
| --- | --- | --- | --- | --- | --- | --- |
| Fig. 6A, red; Tris pH 7.5 | 35 µM casp-6 D179 CT, 20 mM Tris pH 7.5, 120 mM NaCl, 2 mM d-DTT, 5% d-Glycerol, 10% D_2_O | 1D ^1^H | H_2_O | Yes; on H_2_O according to [59] | Centered around H_2_O | 288 |
| Fig. 6A, orange; HEPES pH 7.5 | 27 µM casp-6 D179 CT, 50 mM HEPES pH 7.5, 120 mM NaCl, 2 mM d-DTT, 10% D_2_O | 1D ^1^H | H_2_O | Yes; on H_2_O according to [59] | Centered around H_2_O | 288 |
| Fig. 6A, blue; Phosphate pH 7.4 | 30 µM casp-6 D179 CT, 100 mM phosphate, pH 7.4, 120 mM NaCl, 2 mM d-DTT, 10% D_2_O | 1D ^1^H | H_2_O | Yes; on H_2_O according to [59] | Centered around H_2_O | 298 |
| Fig 6B, red; 0 mM NaCl | 33 µM casp-6 D179 CT, 10 mM HEPES pH 7.5, 0 mM NaCl, 60 µM VEID-CHO, 2 mM d-DTT, 2.5% d-DMSO, 10% D_2_O | 1D ^1^H | H_2_O | Yes; on H_2_O according to [59] | Centered around H_2_O | 293 |
| Fig 6B, orange; 60 mM NaCl | 32 µM casp-6 D179 CT, 10 mM HEPES pH 7.5, 60 mM NaCl, 60 µM VEID-CHO, 2 mM d-DTT, 2.5% d-DMSO, 10% D_2_O | 1D ^1^H | H_2_O | Yes; on H_2_O according to [59] | Centered around H_2_O | 293 |
| Fig 6B, blue; 180 mM NaCl | 37 µM casp-6 D179 CT, 10 mM HEPES pH 7.5, 180 mM NaCl, 60 µM VEID-CHO, 2 mM d-DTT, 2.5% d-DMSO, 10% D_2_O | 1D ^1^H | H_2_O | Yes; on H_2_O according to [59] | Centered around H_2_O | 293 |
| Fig 6B, light blue; 240 mM NaCl | 37 µM casp-6 D179 CT, 10 mM HEPES pH 7.5, 240 mM NaCl, 60 µM VEID-CHO, 2 mM d-DTT, 2.5% d-DMSO, 10% D_2_O | 1D ^1^H | H_2_O | Yes; on H_2_O according to [59] | Centered around H_2_O | 293 |
| Fig. 6C, red; 0 mM BOG | 30 µM casp-6 D179 CT, 100 mM phosphate, pH 7.4, 120 mM NaCl, 2 mM d-DTT, 10% D_2_O | 1D ^1^H | H_2_O | Yes; on H_2_O according to [59] | Centered around H_2_O | 298 |
| Fig. 6C, blue; 3 mM BOG | 64 µM casp-6 D179 CT, 100 mM Phosphate pH 7.4, 120 mM NaCl, 3 mM Unlabeled BOG, 2 mM d-DTT, 10% D_2_O | 1D ^1^H | H_2_O | Yes; on H_2_O according to [59] | Centered around H_2_O | 288 |
| Fig. 6D, red; D179 CT | 31 µM casp-6 D179 CT, 20 mM d-Tris pH 7.5, 120 mM NaCl, 2 mM d-DTT, 10% D_2_O | 1D ^1^H | H_2_O | Yes; on H_2_O according to [59] | Centered around H_2_O | 288 |
| Fig. 6D, orange; D179 CT + Glycerol | 31 µM casp-6 D179 CT, 20 mM d-Tris pH 7.5, 120 mM NaCl, 5% d-Glycerol, 2 mM d-DTT, 10% D_2_O | 1D ^1^H | H_2_O | Yes; on H_2_O according to [59] | Centered around H_2_O | 288 |
| Fig. 6D, blue; FL | 146 µM casp-6 FL C163S, 20 mM d-Tris pH 7.5, 120 mM NaCl, 2 mM d-DTT, 10% D_2_O | 1D ^1^H | H_2_O | Yes; on H_2_O according to [59] | Centered around H_2_O | 288 |
| Fig. 6D, light blue; FL + Glycerol | 146 µM casp-6 FL C163S, 20 mM d-Tris pH 7.5, 120 mM NaCl, 5% d-Glycerol, 2 mM d-DTT, 10% D_2_O | 1D ^1^H | H_2_O | Yes; on H_2_O according to [59] | Centered around H_2_O | 288 |
| Fig. 7 | 190 µM ^13^C-Ileδ1-methyl labeled casp-6 D179 CT, 20 mM d-Tris pH 8.5, 200 mM NaCl, 5% d_6_-glycerol, and 5 mM d-DTT in 100% D_2_O | 2D ^1^H-^13^C HMQC | D_2_O | Yes; on H_2_O according to [60] | Centered around residual H_2_O in ^1^H; ^13^C was calculated using ^1^H reference in NMRPipe. | 308 |
| S2 Fig | 80 µM ^13^C-Ileδ1-methyl labeled casp-6 D179 CT, 20 mM d-Tris pH 8.5, 200 mM NaCl, 5% d_6_-glycerol, and 10 mM d-DTT in 100% D_2_O – Incubated 0 hours at 25 °C | 2D ^1^H-^13^C HMQC | D_2_O | Yes; on H_2_O according to [60] | Centered around residual H_2_O in ^1^H; ^13^C was calculated using ^1^H reference in NMRPipe. | 298 |
| S2 Fig | 80 µM ^13^C-Ileδ1-methyl labeled casp-6 D179 CT, 20 mM d-Tris pH 8.5, 200 mM NaCl, 5% d_6_-glycerol, and 10 mM d-DTT in 100% D_2_O – Incubated 8 hours at 25 °C | 2D ^1^H-^13^C HMQC | D_2_O | Yes; on H_2_O according to [60] | Centered around residual H_2_O in ^1^H; ^13^C was calculated using ^1^H reference in NMRPipe. | 298 |
| S2 Fig | 80 µM ^13^C-Ileδ1-methyl labeled casp-6 D179 CT, 20 mM d-Tris pH 8.5, 200 mM NaCl, 5% d_6_-glycerol, and 10 mM d-DTT in 100% D_2_O – Incubated 16 hours at 25 °C | 2D ^1^H-^13^C HMQC | D_2_O | Yes; on H_2_O according to [60] | Centered around residual H_2_O in ^1^H; ^13^C was calculated using ^1^H reference in NMRPipe. | 298 |
| S2 Fig | 80 µM ^13^C-Ileδ1-methyl labeled casp-6 D179 CT, 20 mM d-Tris pH 8.5, 200 mM NaCl, 5% d_6_-glycerol, and 10 mM d-DTT in 100% D_2_O – Incubated 24 hours at 25 °C | 2D ^1^H-^13^C HMQC | D_2_O | Yes; on H_2_O according to [60] | Centered around residual H_2_O in ^1^H; ^13^C was calculated using ^1^H reference in NMRPipe. | 298 |
| S2 Fig | 80 µM ^13^C-Ileδ1-methyl labeled casp-6 D179 CT, 20 mM d-Tris pH 8.5, 200 mM NaCl, 5% d_6_-glycerol, and 10 mM d-DTT in 100% D_2_O – Incubated 48 hours at 25 °C | 2D ^1^H-^13^C HMQC | D_2_O | Yes; on H_2_O according to [60] | Centered around residual H_2_O in ^1^H; ^13^C was calculated using ^1^H reference in NMRPipe. | 298 |
| S2 Fig | 80 µM ^13^C-Ileδ1-methyl labeled casp-6 D179 CT, 20 mM d-Tris pH 8.5, 200 mM NaCl, 5% d_6_-glycerol, and 10 mM d-DTT in 100% D_2_O – Incubated 72 hours at 25 °C | 2D ^1^H-^13^C HMQC | D_2_O | Yes; on H_2_O according to [60] | Centered around residual H_2_O in ^1^H; ^13^C was calculated using ^1^H reference in NMRPipe. | 298 |
| S2 Fig | 80 µM ^13^C-Ileδ1-methyl labeled casp-6 D179 CT, 20 mM d-Tris pH 8.5, 200 mM NaCl, 5% d_6_-glycerol, and 10 mM d-DTT in 100% D_2_O – Incubated 96 hours at 25 °C | 2D ^1^H-^13^C HMQC | D_2_O | Yes; on H_2_O according to [60] | Centered around residual H_2_O in ^1^H; ^13^C was calculated using ^1^H reference in NMRPipe. | 298 |
| S2 Fig | 80 µM ^13^C-Ileδ1-methyl labeled casp-6 D179 CT, 20 mM d-Tris pH 8.5, 200 mM NaCl, 5% d_6_-glycerol, and 10 mM d-DTT in 100% D_2_O – Incubated 120 hours at 25 °C | 2D ^1^H-^13^C HMQC | D_2_O | Yes; on H_2_O according to [60] | Centered around residual H_2_O in ^1^H; ^13^C was calculated using ^1^H reference in NMRPipe. | 298 |

59. Hwang TL, Shaka AJ. Water Suppression That Works. Excitation Sculpting Using Arbitrary Wave-Forms and Pulsed-Field Gradients. Journal of Magnetic Resonance, Series A. 1995;112(2):275-9. doi: 10.1006/jmra.1995.1047.

60. Schanda P, Kupče Ē, Brutscher B. SOFAST-HMQC Experiments for Recording Two-dimensional Deteronuclear Correlation Spectra of Proteins within a Few Seconds. J Biomol NMR. 2005;33(4):199-211. doi: 10.1007/s10858-005-4425-x.
