# Supplementary material for: An NMR sample preparation case study: Considerations for the self-destructive protease caspase-6
Source: PLoS One. 2025 Nov 21;20(11):e0337291. doi: 10.1371/journal.pone.0337291 (PMC12637907; doi:10.1371/journal.pone.0337291)
Supplement: S1 Protocol — A step by step procedure of protein expression, purification, and buffer exchange for isotopically-labeled casp-6 NMR samples. (DOCX) [file pone.0337291.s006.docx]

**Growth and Overexpression**

This section will cover the necessary chemicals needed for preparing minimal media for labeled growth, the chemicals needed for different labeling schemes, and the protocols for media preparation and growth itself. Due to the intrinsic nature of proteins to not express as well in minimal media, I would encourage going through unlabeled optimization to find the amount of minimal salts (2x M9 salts concentration for casp-6), induction OD_600_ (1.0-1.1 for casp-6), induction temperature (20 °C for casp-6), and induction time (18-22 hrs for casp-6).

Note that you can also modify other parameters such as growth temperature, the amount of glucose, or using different vitamins and minerals.

Materials Needed for 1 L 2x M9 Minimal Media

| REAGENT | Mass | VOLUME of Stock | STOCK CONC. | FINAL CONC. |
| --- | --- | --- | --- | --- |
| Na_2_HPO_4_ | 13.56 g | - | - | 13.56 g/L |
| KH_2_PO_4_ | 6 g | - | - | 6 g/L |
| NaCl | 1 g | - | - | 1 g/L |
| ddH_2_O | - | 943.5 mL | - | - |
| Glucose | 2 g | - | - | 2 g/L |
| NH_4_Cl | 1 g | - | - | 1 g/L |
| Ampicillin | 100 mg | - | - | 100 mg/L |
| ddH_2_O | - | 25 mL | - | - |
| Vitamin Mix | - | 10 mL | 1 mg/mL (each vit.) | 10 µg/mL (each vit.) |
| Biotin | - | 1 mL | 10 mg/mL | 10 µg/mL |
| MgSO_4_ | - | 2 mL | 1 M | 2 mM |
| CaCl_2_ | - | 1.5 mL | 100 mM | 150 µM |
| Na_2_SO_4_ | - | 15 mL | 1 M | 15 mM |
| FeCl_3_ | - | 2 mL | 100 mM | 200 µM |

*Recipes for each of the stock solutions can be found at the bottom of this section

Supplements for Isotopic Labeling

| Labeling Scheme | Reagent/Precursor | Mass | Final Conc. |
| --- | --- | --- | --- |
| Uniform ^13^C | Glucose | 2 g | 2 g/L |
| Uniform ^15^N | NH_4_Cl | 1 g | 1 g/L |
| ^13^C-Ile δ1 Me | 2-ketobutryate | 60 mg | 60 mg/L |
| ^13^C-Leu δ Methyls and Val γ Methyls | 2-ketoisovalerate | 80-120 mg | 80-120 mg/L |

*Note that there are numerous other labeling schemes that can be used as well. For more information on specific methyl labeling including stereospecificity refer to the paper below. Additionally, deuteration can be incorporated by using fully/partially deuterated reagents/precursors, deuterated glucose, and growing in D_2_O (note that the deuteration protocol is different from this document due to D_2_O’s toxicity).

Kerfah R, Plevin MJ, Sounier R, Gans P, Boisbouvier J. Methyl-specific isotopic labeling: a molecular tool box for solution NMR studies of large proteins. Current Opinion in Structural Biology. 2015;32:113-22.

PREPARATION OF 1 L 2x M9 MINIMAL MEDIA (non-D_2_O)

1. Dissolve the Na_2_HPO_4_, KH_2_PO_4_, and NaCl in 800 mL Nanopure H_2_O
2. Use a pH strip to ensure that the pH is at ~7.4
   1. Use concentrated HCl or 5 M NaOH if adjustments are needed
3. Add an additional 143.5 mL Nanopure H_2_O to the salts and continue stirring to mix
4. Transfer 2x M9 salts into 1.4 L Fernbach flask and autoclave to sterilize

***NOTE*: If making more than 1 L (recommended 4-8 L for casp-6) a 10x M9 salts stock can be prepared, have pH adjusted, autoclaved, and stored for quick preparation of 2x M9 salts. In this case 200 mL of the 10x M9 stock as well as 743.5 mL of nanopure water can be added to each 1.4 L Fernbach flask prior to autoclaving.**

1. In a separate beaker combine the remaining nutrients, vitamins, and minerals in the following order (MAKE SURE TO USE THIS MIX WITHIN THE DAY)
   1. Glucose, NH_4_Cl, Ampicillin, 25 mL H_2_O, Vitamin Mix, Biotin, MgSO_4_, CaCl_2_, NaSO_4_, and LASTLY FeCl_3_, if solution appears to have precipitate or is a dark orange, adjust with concentrated HCl (to redissolve the FeCl_3_) until solution is a transparent and pale yellow color
2. If making more than 1 L and there is more than 56.5 mL, this vitamin and mineral mix can be filter sterilized into a bottle. If preparing only 1 L, this mix can be left in the beaker and dispensed into the M9 salts later through a syringe-driven filter

GROWTH PROTOCOL

NOTE: The timing for inoculation, addition of precursors, induction, and harvesting can be non-ideal (e.g. middle of the night) so I suggest the schedule below.

Day 1:

1. Around 4-4:30 pm prepare 4 seed cultures of LB in volumes of 4 mL, 5 mL, 6 mL, and 7 mL at a sterile bench near an open flame.
   1. Each seed culture should be made up of the 4 mL, 5 mL, 6 mL or 7 mL of LB media, 1/1000^th^ the respective volume of 100 mg/mL Ampicillin (for example 4 µL for 4 mL), and a single colony from a fresh LB Agar plate.
2. Begin shaking the seed cultures at 37 °C.
3. After 3-3.5 hours, check the OD_600_ of each seed culture. We are seeking at least one culture to have an OD_600_ at ~0.6).
4. Again, at a sterile bench by an open flame, either filter the vitamin and mineral mix or dispense the already filtered mix into the 2x M9 salts.
5. Around 7:30-8 pm inoculate each 1L culture with 250 µL of the OD_600_ 0.6 seed culture and begin shaking at 30 °C and 200 rpm overnight.

Day 2:

1. The next morning, check the OD_600_ to ensure that the cells have not overgrown.
2. Once the cells reach an OD_600_ of ~0.7-0.75, weigh out the needed amino acid precursor (if using) and add to the culture in a solid or concentrated (in nanopure H_2_O) form.
   1. Caspase-6 cultures typically reach 0.7-0.75 around 11-11:30 am.
3. One hour after the addition of the precursor, the OD_600_ should be around 1.0-1.1. Induce the culture with IPTG to a final concentration of 1 mM (1 mL of 1 M for a 1 L culture) and adjust the temperature of the shaking incubator to 20 °C.
   1. If the OD_600_ is not quite to the optimal density, the culture can be left to grow for another 15-30 minutes, but do not wait too long after addition of the precursor for induction to prevent loss of isotopic signal.
4. Around 12-12:30 pm, the culture should be left to induce protein expression shaking at 20 °C for ideally 18-20 hours

Day 3:

1. The next morning around 8 am the culture should be harvested by centrifugation in 1 L bottles in the SLC-4000 rotor at 4,696 x g for 10 min at 4 °C.
   1. It is recommended to start cooling down the centrifuge 30 min prior to harvesting.
2. The cell pellet should be transferred to a 50 mL conical tube, mass should be recorded and the cells should be frozen at -80 °C.

RECIPES FOR STOCK SOLUTIONS

**Vitamin Mix**

- 25 mg Thiamine
- 25 mg Nicotinic acid
- 25 mg Calcium pantothenate

Dissolve in 25 mL ddH_2_O and filter sterilize. Store wrapped in foil at 4 °C.

**Biotin**

- 25 mg Biotin

Dissolve in 2.5 mL ddH_2_O with 1-2 drops 1M NaOH. Filter sterilize and store at 4 °C.

**MgSO_4_**

- 24.07 g MgSO_4_ (anhydrous)

Dissolve in 200 mL ddH_2_O and filter sterilize. Store at room temperature.

**CaCl_2_**

- 2.94 g CaCl_2_ · 2 H_2_O or 2.22 g CaCl_2_ (anhydrous)

Dissolve in 200 mL ddH_2_O and filter sterilize. Store at room temperature.

**Na_2_SO_4_**

- 14.22 g Na_2_SO_4_

Dissolve in 100 mL ddH_2_O and filter sterilize. Store at room temperature.

**FeCl_3_**

- 81.1 mg FeCl_3­_

Dissolve in 5 mL ddH_2_O with 1-2 drops concentrated HCl and filter sterilize. Store away from metals, bases, and oxidizing materials in a cool dry place.

**Purification**

Materials

***NOTE*: Buffers can either be made from solid or stock solutions, but should have pH adjusted with a pH probe, vacuum filtered through a 0.22 µm filter to sterilize, and degassed by stirring under vacuum for ~45 minutes**

Lysis Buffer – 50 mM Tris pH 8.5, 300 mM NaCl, 5% Glycerol, 50 mM Imidazole

Elution Buffer– 50 mM Tris pH 8.5, 300 mM NaCl, 5% Glycerol, 1 M Imidazole

Ni Column – Cytiva HisTrap HP 5 mL

Nanopure H_2_O

Isopropanol

LYSING THE CELLS via Microfluidizer

1. Turn on the floor centrifuge and begin cooling to 4 °C with the SS-34 rotor.
2. The cell pellet should be removed from the -80 °C freezer, the 50 mL conical tube should be filled to ~35 mL with lysis buffer and placed in a 37 °C water bath for 20 minutes.
3. Close off the drain tube for the microfluidizer and begin filling the container around the coil with ice to ensure everything is properly cooled (I usually push ice into the coil as well to slow the melting process).
4. Flip the pressure lever on the pipe against the wall and bring the pressure up to 9,000 psi.
5. To prepare the microfluidizer, run 900 mL of water and 300 mL of lysis buffer through the instrument taking care not to introduce air into the system.
6. Remove the thawed cell pellet from the water bath and shake/vortex to completely homogenize the cells.
7. Turn the pressure up to 15,000 psi and pass the cell suspension through the microfluizier 5 times to ensure complete cell lysis.
   1. Note that each time the cells pass through only takes ~3 pumps of the microfluidizer.
8. Pour ~15 mL lysis buffer into the bottom of the fill tube and run this through the microfluidizer to collect any cells left in the tubing.
9. Balance the lysed cells in the SS-34 centrifuge tubes and begin spinning down at 15,000 rpm and 4 °C for 45 minutes.
10. To clean the microfluidizer, carefully remove the glass fill tube, rinse with 20% bleach in sink and wash with soapy water. Finally, rinse with DI water.
11. Replace the fill tube on the microfluidizer and at 9,000 psi run three fill tubes of water (~900 mL) and 2/3 a fill tube of isopropanol (~200 mL), leaving about 1/3 of a fill tube (~100 mL) of isopropanol in the tube.
12. Cover the fill tube with parafilm to prevent evaporation, release the pressure, flip the pressurized air lever against the wall, and drain the melting ice into the bucket on the floor.
13. Mist the area around the microfluidizer and balancing bench with 70% EtOH.

PREPARING THE ÄKTA™ pure

1. Label all the bottles and a deep well plate that will be needed.
   1. NiFT, Ni Wash, Lys (conical bottle), Ni Fractions
2. Thoroughly rinse and place each line and outlet in the respective bottles/buffers
   1. A1 🡪 Lysis Buffer, B1 🡪 Elution Buffer, S1 🡪 Lysis Buffer, Buffer Chase 🡪 Lysis Buffer
   2. Outlet 1 🡪 NiFT, Outlet 2 🡪 Ni Wash
3. Prime each A and B inlet by selecting the appropriate inlet position on the inlet valves in the System control window and drawing ~25 mL of buffer through the left pump head from the inlet’s corresponding pump.
   1. This needs to be done in reverse alphabetical order and if multiple A lines and multiple B lines are being used then the lines should also be primed in reverse numerical order.
      1. B2, B1, A2, A1
4. Prime the sample inlets and buffer chase line similarly, this time drawing ~15 mL of buffer through
   - 1. S2, Buffer Chase, S1
5. Upon starting the system, a rapid increase in pressure may be due to air and the pumps may need to be purged by drawing more buffer while system is running. (Refer to the ÄKTA™ pure manual for instructions).
6. Use pump washes to prepare each pump for the first buffer that will be used with each of them.
   1. This is done in the system pumps and sample pump controls in the system control window
      1. B1 pump wash, A1 pump wash, S1 pump wash
7. Now attach the columns using WET CONNECTIONS and a flow rate of ~2 mL/min through A1
   1. Ni column 🡪 Column position 1

STARTING THE Ni PURIFICATION

***NOTE*: Detailed text instructions for the different protocols used as well as images of the method editor window can be found in S1 Data under “Purification Methods.”**

1. If the lysed cells are still being centrifuged when preparing the ÄKTA™ system is prepared for the purification, the column can begin being equilibrated and the protocol “C6 Nickel Affinity with Quick Wash and 1 M Imidazole” can be used, otherwise skip this step and use the protocol “C6 Nickel Affinity with Quick Wash and 1 M Imidazole with Equilibration.”
   1. To manually equilibrate the Ni Column, start a flow of 5 mL/min through A1 and set the column valve position to position 1.
   2. Monitor the absorbance (UV (mAU)) until it stabilizes or until 10 column volumes (CVs) of lysis buffer have been run.
2. Add the lysate from the centrifugation into the conical bottle “Lys”
3. Transfer line S1 to the lysate and ensure the system is in the “Ready” state which can be done by pressing the “Stop” (■) button in the System Control window.
4. Place the deep well plate “Ni Fractions” in the holder matching up the A1 corner and place in the fraction collector with the notched side facing out.
5. Start the “C6 Nickel Affinity with Quick Wash and 1 M Imidazole with Equilibration” protocol if yet to equilibrate the column or the “C6 Nickel Affinity with Quick Wash and 1 M Imidazole” protocol if the column has already been equilibrated
   1. Upon opening the method in the System Control window ensure that the 96 deep well plate is recognized, and the parameters outlined in the ÄKTA™ PDF for Ni Purification are correct (ignore equilibration parameters for “C6 Ni Affinity” protocol).
   2. These protocols consist of the following steps (excluding column equilibration).
      1. Sample Application
         1. Full sample volume through S1 at a flow rate of 3 mL/min with NiFT through Outlet 1
      2. Column Wash
         1. Wash with Lysis buffer for 10 CVs at 5 mL/min collecting Ni wash flow-through through Outlet 2.
      3. Elution
         1. Elution using 73.5% Lysis Buffer and 26.5% Elution Buffer (300 mM Imidazole) for 5 CVs at 1 mL/min collected in 0.5 mL fractions in a 96 deep-well plate.
      4. Column Wash with iterative cycles of Lysis Buffer and Elution Buffer.
6. During the purification monitor the UV, pH, and Conductivity and make sure that there is enough buffer throughout the duration of the run.

PREPARING SDS-PAGE GEL SAMPLES AND RUNNING THE PURIFICATION GEL

1. The samples that should be analyzed by reducing SDS-PAGE on a 16% gel are as follows
   1. Protein Marker (PM), Cell Debris (CD), Lysate (Lys), Nickel Flow-Through (NiFT), Ni Wash, and individual chosen Ni fractions.
2. First remove an aliquot of 1 M DTT and 3x SDS Loading Dye from the -20 °C freezer to begin thawing.
3. To prepare the CD sample, use a pipette tip to scoop a small amount of the cell pellet from one of the tubes from spinning down and resuspend it in 1 mL of lysis buffer.
   1. The CD gel sample will be made up of 30 µL of this suspension and 10 µL of prepared 3x SDS Loading Dye.
4. The Lys and NiFT samples also need to be diluted due to their high protein content, so each should have 20 µL mixed with 60 µL of lysis buffer prior to making the gel sample.
5. Prepare the SDS Loading Dye by mixing 1 part 1 M DTT with 7 parts 3x SDS Loading Dye (made in house).
6. Each gel sample should be made up of 1 part prepared loading dye and 3 parts sample
   1. Example: Generally we will do 10 µL Dye and 30 µL Sample
7. Once the Ni-affinity protocol has finished, use the UV curve to choose which fractions to analyze by reducing SDS-PAGE.
   1. It is not necessary to analyze every fraction; often doing every other, focusing on peaks is sufficient.
8. 4 µL of the protein 10-200 kDa Molecular Weight Marker (New England Biolabs) and 8 µL of every other sample should be loaded onto a 16% gel.
9. Run the gel for 55 minutes at 170 V.
10. If planning to concentrate fractions, the spin filter can be equilibrated while the gel is running.
11. Once the gel is complete, develop with fixing solution, Coomassie blue stain, then destaining solution.
12. Once the gel is developed, choose which fractions to pool and aliquot, concentrate, exchange or use for another experiment based on concentration and purity
13. Concentration can be measured by measuring A280 on the nanodrop and calculating with the extinction coefficient (for example 25,900 M^-1^cm^-1^ for C6 ΔN D179 CT)
    1. The blank used for this should be made up of the same percentages Lysis buffer and Ni-Elution buffer that the protein eluted at
    2. Note that the high imidazole concentration can often make it difficult to estimate concentration by A280

CLEANING UP

1. If protein was successfully purified and is not in the FTs or washes, all those bottles can be emptied and washed in the dishwasher.
   1. NiFT should be bleached briefly to ensure to kill any remaining cells.
2. Cell pellets and CD, Lys, and NiFT dilutions should be placed in biohazard waste to be autoclaved
3. 96-deep well plates need to be hand washed with warm water, soapy water, rinsed thoroughly with warm water, and then rinsed thoroughly with DI water before being left to dry.
4. Rinse all the lines and place them back into their respective degassed ddH_2_O bottles.
5. Remove the columns with water running through the column position that is being handled to ensure wet connections are being made.
6. Ensure the waste container is not almost full and replace the black spout in the fraction collector with a small 50 mL cup.
7. Before running the system CIP reprime the system pump line S1 as was done at the beginning with the degassed ddH_2_O.
   1. This is to remove the air from these line that was introduced during sample application.
8. Run the custom system CIP making sure that all the lines, outlets, and column positions that were used are checked.

**Buffer Exchange**

PD-10/Desalting Columns

This gravity flow protocol was determined to be the most efficient method of buffer exchange for casp-6.

NMR Buffer: 20 mM d-Tris pH 8.5, 200 mM NaCl, 5% d_6_-glycerol, 10 mM d-DTT in 100% D_2_O

1. Prepare the buffer by adding solid amounts of the deuterated reagents and dissolving in D_2_O followed by testing the pH of the solution with pH paper.
2. Equilibrate the PD-10 desalting column according to the manufacturer’s instructions by flowing 20 mL through the Sephadex G-25 bedding.
3. Chosen Ni Elution fractions should be concentrated to 2.5 mL for a PD-10 column and loaded onto desalting column using gravity.
4. Protein should then be eluted using 3.5 mL and 5-10 drop (~170 µL) fractions collected in a 96-well plate.
5. Fractions can then be analyzed on an SDS-PAGE gel to test purity and concentration.
6. Concentration of pools can be measured by absorbance at 280 nM and aliquoted at 170 µL prior to freezing at -80 °C.
7. Individual samples can then be thawed for NMR experiments in a 3 mm tube.
