# Supplementary material for: An NMR sample preparation case study: Considerations for the self-destructive protease caspase-6
Source: PLoS One. 2025 Nov 21;20(11):e0337291. doi: 10.1371/journal.pone.0337291 (PMC12637907; doi:10.1371/journal.pone.0337291)
Supplement: S1 Raw Images — Original uncropped images of the caspase-6 purification and exchange gels. (PDF) [file pone.0337291.s008.pdf]

S1_raw_images

All SDS-PAGE gels were imaged on a Bio-Rad ChemiDoc MP Imaging System following staining with Coomassie Blue.

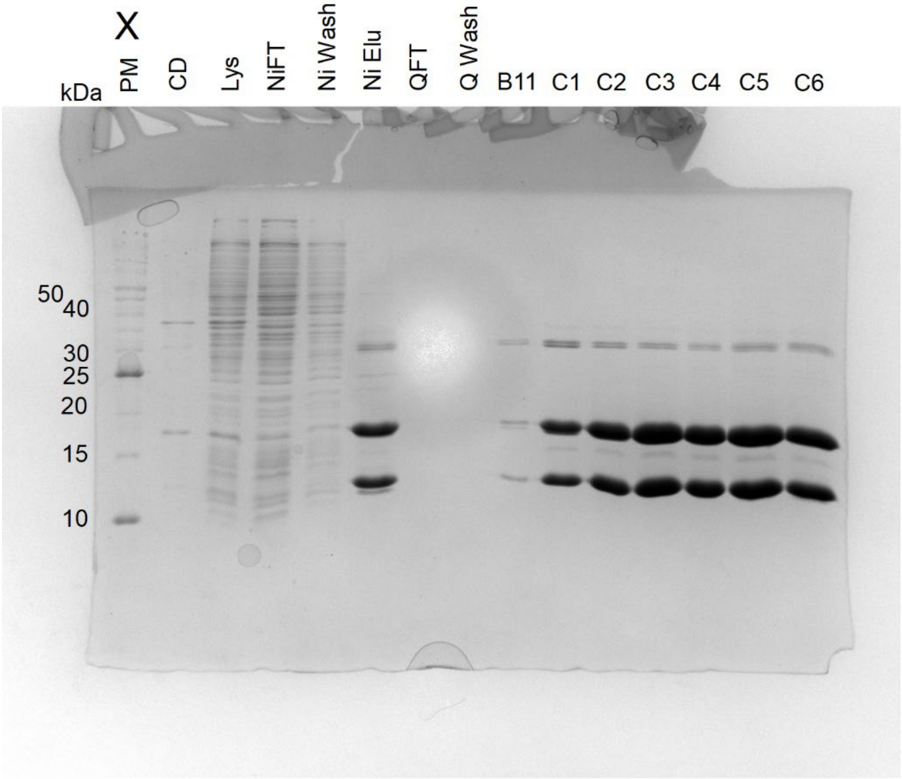

Figure 3C - Nickel-Affinity and Anion Exchange Purification Gel

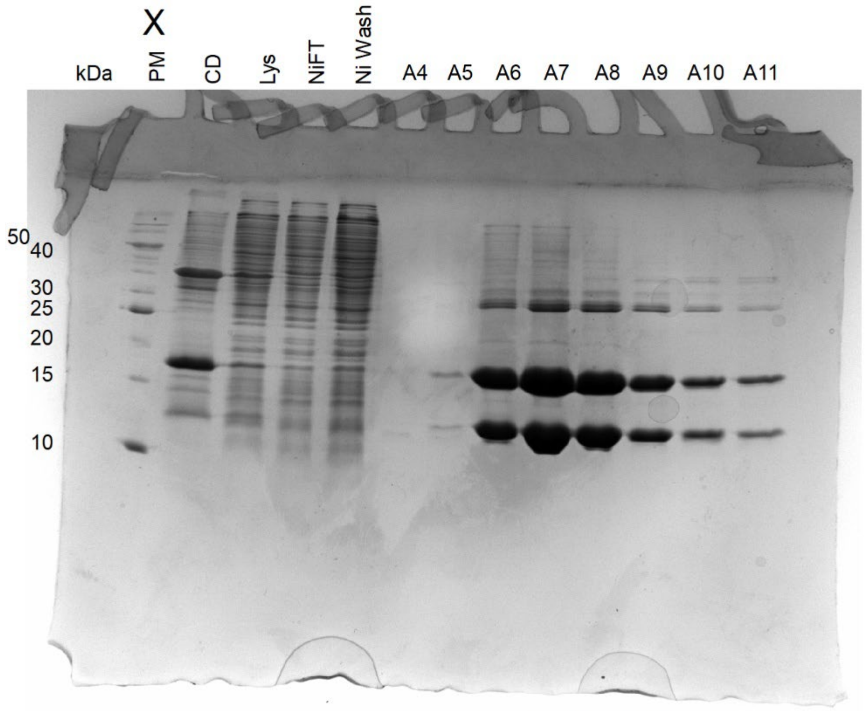

Figures 3D and 7 - Nickel-Affinity Purification Gel

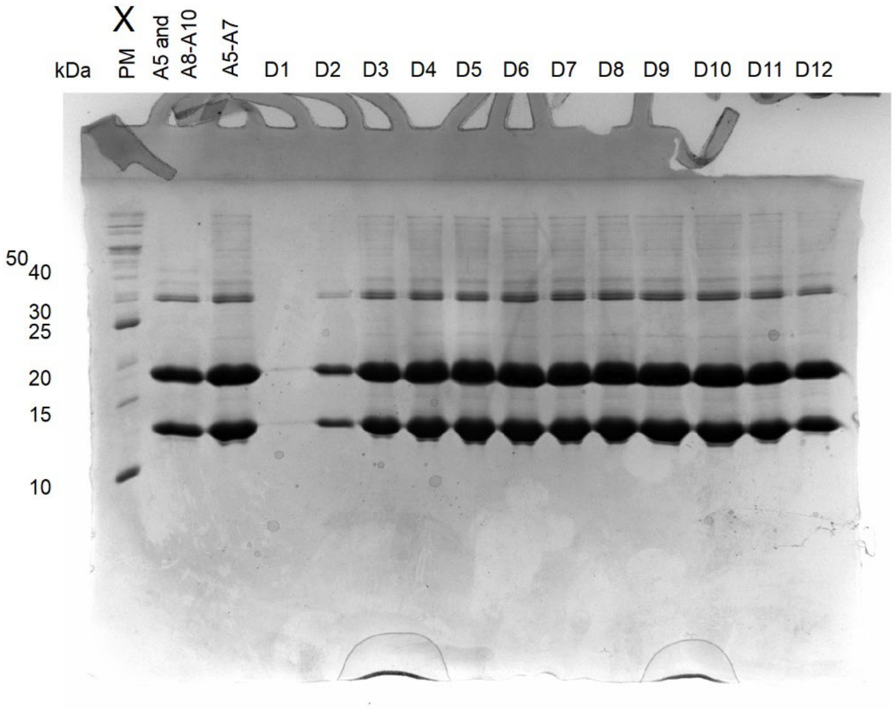

Figures 3E and 7 - Post-Buffer Exchange Gel

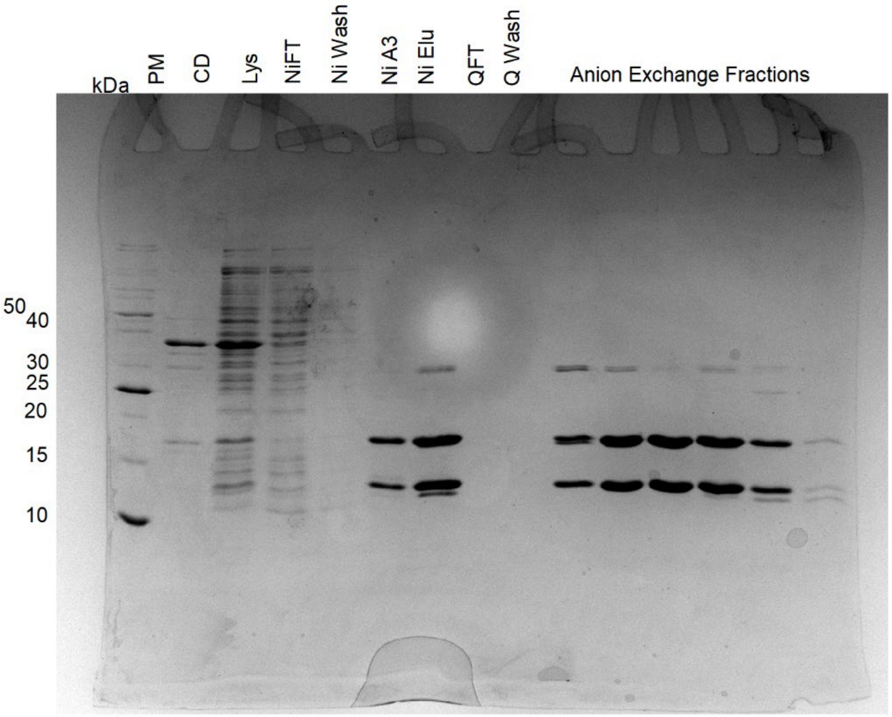

Figure S1 (top left) - 2x M9 Minimal Media OD600 = 1.1 Purification Gel

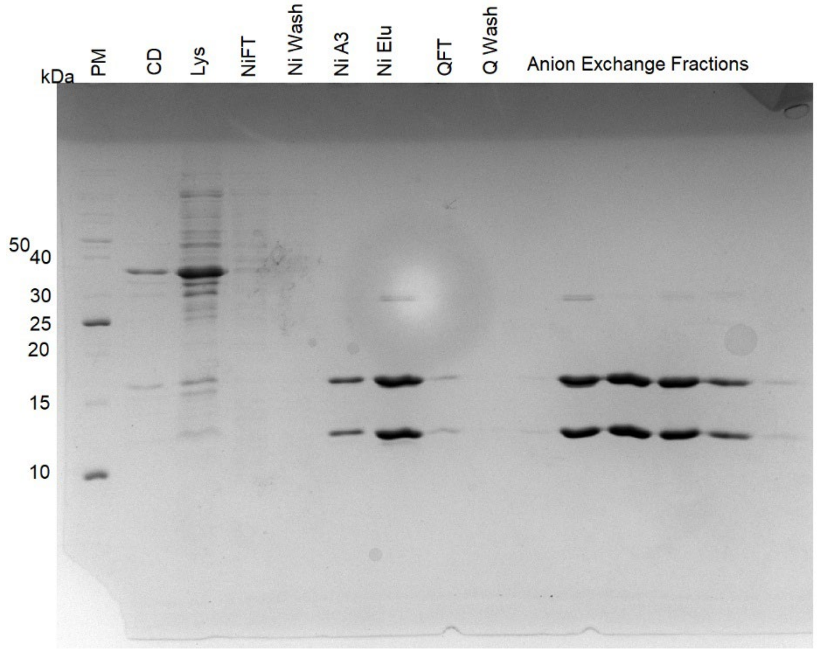

Figure S1 (top right) - 2x M9 Minimal Media OD600 = 1.1 Purification Gel

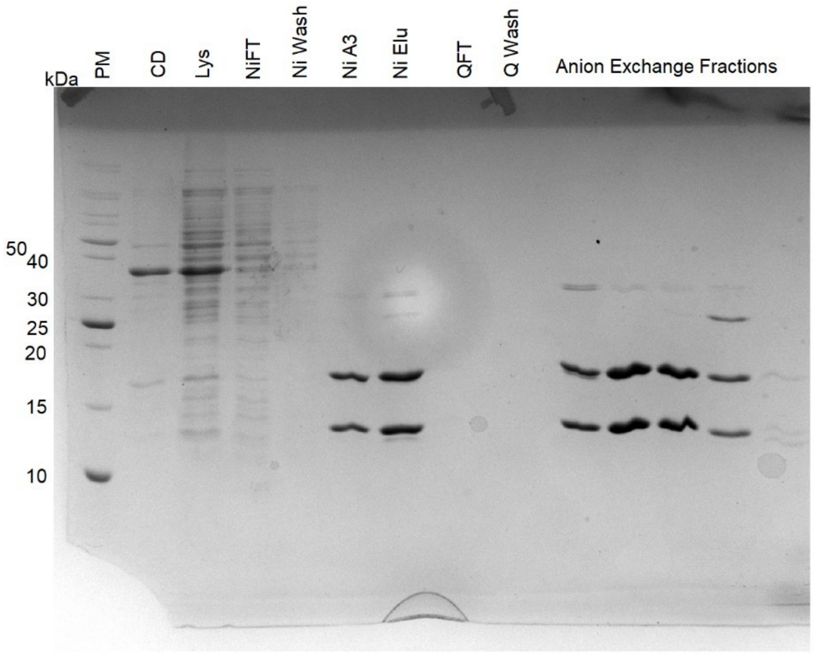

Figure S1 (bottom left) - 2x M9 Minimal Media OD600 = 1.6 Purification Gel

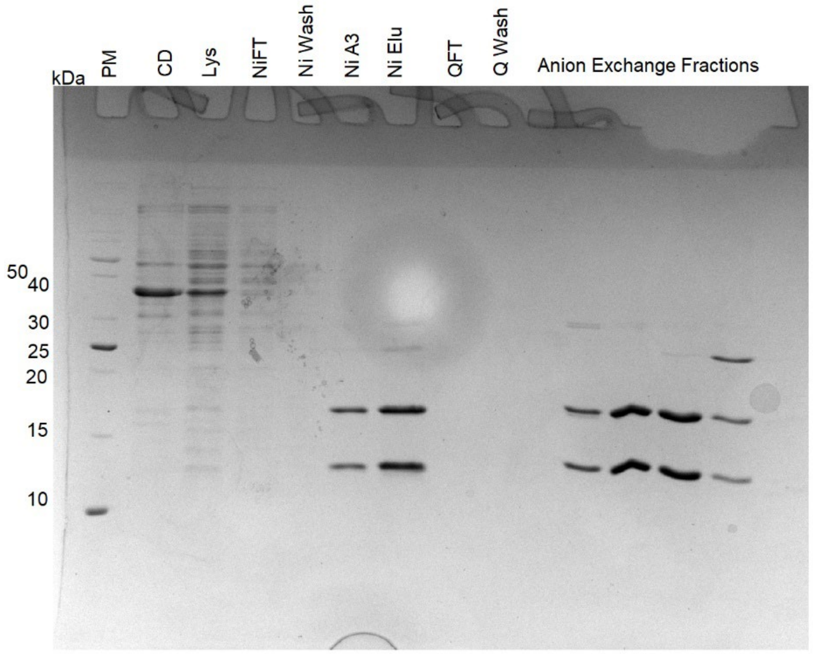

Figure S1 (bottom right) - 2x M9 Minimal Media OD600 = 2.0 Purification Gel
