# Supplementary material for: An NMR sample preparation case study: Considerations for the self-destructive protease caspase-6
Source: PLoS One. 2025 Nov 21;20(11):e0337291. doi: 10.1371/journal.pone.0337291 (PMC12637907; doi:10.1371/journal.pone.0337291)
Supplement: Attachment — Submitted filename: An NMR Sample Preparation Case Study.pdf [file pone.0337291.s009.pdf]

An NMR Sample Preparation Case Study: Considerations for the Self-Destructive Protease Paper Review and Comments by : Dr SAI KUMAR BADAM

1. The introduction **is** scientifically solid and well-supported and the introduction does an excellent job of situating caspase-6 (casp-6) in the context of the broader caspase family, covering structural, functional, and pathological aspects.
2. **Results:**

   Caspase-6 can be satisfactorily overexpressed in *E. coli* in 2x M9 minimal media:

Strengths

The section clearly moves from the problem (need for isotopically labeled casp-6) → method (growth in rich vs minimal media) → variable testing (M9 vs 2×M9, induction OD) → conclusion (optimal growth conditions).

Growth media composition, inoculation strategies, temperature, IPTG concentration, and OD600 ranges are clearly stated, making replication possible.

Mention of previous studies ([50, 51]) to justify testing 2×M9 provides good context for why this variable was examined.

Areas for Improvement

Several sentences repeat the same idea in slightly different forms (e.g., higher yield in minimal media is explained in multiple places).

Figures (e.g., Fig. 2B, Fig. 2D) are referenced, but the reader must infer trends from the text. Briefly **state the key numeric differences or statistical significance** to make the results stand alone from the figures.

The final sentence does give the optimal condition (2×M9, OD600 = 1.1), but this could be **highlighted more strongly** as the experimental takeaway.

Example:

Based on these findings, the optimal expression condition for isotopically labeled casp-6 in E. coli is growth in 2×M9 minimal media with induction at OD600 = 1.1 using 1 mM IPTG at 20 °C for 18 h.

3. **Elution concentration improved by optimizing flow rates and 1**50 **gradients during purification:**

Strengths

You start with a well-defined problem (sample loss during concentration when <40 µM) and link it directly to the need for optimizing purification parameters.

You reference your lab's and others' previous purification strategies, making it clear this is an incremental but meaningful improvement.

Flow rate, elution profile, and fraction volume are discussed in a sequence that mirrors how a reader might troubleshoot the same issue.

You explicitly tie Protocol 4's conditions (230 mM NaCl step elution + 1 mL/min + 0.5 mL fractions) to the desired outcome (higher concentration and minimal loss during buffer exchange).

Areas for Improvement

The section is heavy on procedural parameters (flow rates, NaCl concentrations, column types) in one long paragraph. Splitting it into **two smaller paragraphs**—(1) rationale & design changes, (2) results & optimal conditions—would improve readability.

Some sentences feel more like *methods* than *results*. For example:

"Lower flow rates allowed more contact time between the elution buffer and the ion exchange resin giving a more concentrated sample…"
could be rewritten as a result first:
"Reducing the flow rate from X to 1 mL/min increased the protein concentration in the eluted fractions (Fig. 3B), likely due to longer resin-buffer contact time."

The improvement is well described qualitatively, but there are **no actual concentration or yield numbers**. Even approximate values (e.g., "final elution concentration increased from ~25 µM to ~60 µM") would strengthen the claim and allow readers to gauge the benefit.

4. **pH impacts casp-6 purification and yield**

Strengths

You start by clearly explaining why low pH is desirable for NMR (reduced amide proton exchange), which immediately grounds the pH experiments in their functional relevance.

The decision to stick with pH 8.5 buffers and simplify the workflow (skipping anion exchange) is clearly stated and logically justified.

Areas for Improvement

This section mentions "significantly lower yields" but provides no yield values or percentages. Including even approximate numbers (e.g., "yields dropped by ~50%") would make the impact clear.

The logic chain from "low pH purification fails" → "high pH works" → "skip anion exchange" is correct, but the transitions could be made smoother by explicitly connecting the findings between each step.

5. **<u>Buffer Exchange by desalting column provides the most efficient recovery:</u>**

Strengths

The text explains why deuterated buffers are necessary for NMR and why buffer exchange is an unavoidable step.

Provides meaningful parameters (pH, buffer composition, dilution factor, recovery percentages, column type, sample volume, etc.) that allow reproducibility.

Connects changes in pH, glycerol, and DTT to observed precipitation or recovery differences, with mechanistic reasoning.

Areas for Improvement

Many sentences run 3–4 lines long, making them harder to follow. Breaking them into shorter, concise statements will improve clarity.

For example, lines 222–229 could be split into two or three sentences to separate findings on *column size* from findings on *sample volume*.

The section moves back and forth between *method comparisons* and *factor influences*. It might read more clearly if you present:

Method comparison results (centrifugal filter vs. desalting column vs. dialysis).

Optimization trials for desalting column (volume, size, pH, glycerol, DTT).

Final recommended conditions. Phrases like "much less casp-6 was lost" could be supported with numerical recovery percentages for precision.

The phrase "significant casp-6 loss to precipitation" would be stronger with quantified loss (e.g., “>40% loss”).

The statement on precipitation due to pH change and isoelectric point crossing is strong but could be made crisper:
"Loss of protein was likely due to precipitation near its isoelectric point, resulting in instability or increased non-specific binding to Sephadex resin."

6. <u>Tris buffer is optimal for recovery of functional casp-6 and NMR data collection:</u>

Strengths

You connect buffer optimization directly to NMR signal quality and functional enzyme activity, which is exactly the right framing for a methods/results section.

You used both VEID-amc enzymatic assays and ^1H NMR spectra to evaluate buffer conditions, which reinforces confidence in your conclusions. Inclusion of NaCl titrations, pH variations, and multiple buffer additives shows thorough exploration of variables.

You explicitly note why certain options (like deuterated BOG) were rejected despite minor improvements, which gives the reader insight into cost–benefit considerations in experimental design.

The observation that FL C163S has sharper amide peaks while D179 CT has better methyl dispersion is valuable for others planning similar experiments.

Weaknesses / Points to Clarify

The title “Tris buffer is optimal…” is misleading because your enzyme assay shows *HEPES* gave higher activity, and Tris only stands out for NMR spectral quality. You might want to retitle to something like
"Balancing enzyme activity and spectral quality: Tris buffer enables optimal casp-6 NMR".

Some figure references (like Fig. 6B) describe "no notable effect" but the visual interpretation might not be obvious to all readers; a short quantitative description (e.g., “<5% variation in peak height”) could help.

7. Summary and Conclusions:

Strengths

You connect buffer optimization directly to NMR signal quality and functional enzyme activity, which is exactly the right framing for a methods/results section.

Inclusion of NaCl titrations, pH variations, and multiple buffer additives shows thorough exploration of variables.

You explicitly note why certain options (like deuterated BOG) were rejected despite minor improvements, which gives the reader insight into cost–benefit considerations in experimental design.

The observation that FL C163S has sharper amide peaks while D179 CT has better methyl dispersion is valuable for others planning similar experiments.

Weaknesses / Points to Clarify

The title “Tris buffer is optimal…” is misleading because your enzyme assay shows *HEPES* gave higher activity, and Tris only stands out for NMR spectral quality. You might want to retitle to something like
"Balancing enzyme activity and spectral quality: Tris buffer enables optimal casp-6 NMR".

Some results (e.g., NaCl concentration impact) are discussed twice in slightly different ways, which could be condensed for better flow.

Right now, the enzyme assay results and NMR spectral assessments feel somewhat separated. A linking sentence that explicitly states how functional stability trends matched (or didn't) with NMR signal quality would help the reader.

Some figure references (like Fig. 6B) describe "no notable effect" but the visual interpretation might not be obvious to all readers; a short quantitative description (e.g., "<5% variation in peak height") could help.

8. Materials and Methods:

Strengths:

Procedures are described with sufficient detail to allow replication, including media compositions, induction conditions, and purification steps.

The section follows a natural progression from DNA constructs to expression, purification, activity assays, and finally NMR analysis.

Specifics such as centrifugation speeds, temperatures, buffer compositions, column types, and instrument parameters are well included.

Inclusion of software and statistical methods shows rigor in data handling.

Suggestions for Improvement:

Use consistent spacing between numbers and units (e.g., "100 µg/mL" instead of "100 µg / mL").

Spell out abbreviations at first mention if not common, e.g., "OD600 (optical density at 600 nm)".

Standardize temperature formatting: use "20 °C" consistently (you already do well here).

"The cell pellet was then frozen and stored at -80 °C prior to purification" is repeated after different growth conditions — you can state this once after describing all expression protocols.

In line 426, you wrote "100 µg/mL ampicillin for all caspase-6 constructs." Then in line 432, "100 mg/L ampicillin." Though equivalent, use one unit consistently (preferably µg/mL in microbiology context).

When listing buffer components (lines 430-434), consider using a table or bullet list in supplementary materials for clarity if journal allows.

In line 466: the use of "linear gradient to 15% buffer B" — specify if this means 15% v/v or something else for precision.

The NMR methods are detailed, but consider briefly explaining acronyms like HMQC and SOFAST for broader accessibility.

Mention the rationale for choosing specific constructs for different experiments if relevant (e.g., why 140 µM for FL vs 30-70 µM for D179 CT).

Clarify if temperature variations between 20 °C and 35 °C were for specific purposes or instrument constraints.

You mention multiple R packages and correction methods, which is excellent. Consider briefly mentioning the rationale for choosing Benjamini-Hochberg correction over others.

If the Methods section is too long for the main text, some of the detailed buffer recipes and NMR parameters might be moved to Supplementary Materials with a brief overview retained here.

Summary:

Your **Materials and Methods** section is very solid and well-written. Minor tweaks for consistency, clarity, and readability will polish it further and improve accessibility for diverse readers. Including rationale for some parameter choices would strengthen the scientific narrative.
