# Supplementary material for: An NMR sample preparation case study: Considerations for the self-destructive protease caspase-6
Source: PLoS One. 2025 Nov 21;20(11):e0337291. doi: 10.1371/journal.pone.0337291 (PMC12637907; doi:10.1371/journal.pone.0337291)
Supplement: S1 Data — Data are categorized by its respective main text figure. Files are titled with a description of contents. Exported text instructions and images for optimized purification protocols are in the Purification Methods folder. (ZIP) [file pone.0337291.s007.zip › S1_Data/Purification Methods/C6 Nickel affinity With Equilibration With Quick Wash and 1 M Imidazole.pdf]

Text instructions

Main method:

```
0.00   Base: CV, Vc=5.027 {ml}, (HisTrap HP, 5 ml)#Column type
0.00   Phase: Method Settings
  0.00   Base: SameAsMain
  0.00   Alarm pre column pressure: Enabled, (0.50)#Pre column pressure
  limit {MPa}, 0.00 {MPa}
  0.00   Alarm delta column pressure: Enabled, (0.30)#Delta column pressure
  limit {MPa}, 0.00 {MPa}
  0.00   Noise reduction UV: (2.5)#UV averaging time {sec}
  0.00   Injection valve: Manual load
  0.00   Column position: (1)#Column position, Down flow
  0.00   pH valve: (In-line)#pH cell, (In-line)#Flow restrictor
  0.00   Inlet A: (A1)#Inlet A
  0.00   Inlet B: (B1)#Inlet B
  0.00   Alarm inlet A air sensor: Enabled
  0.00   Alarm sample inlet air sensor: (Enabled)#Air sensor alarm on
  sample pump
  0.00   System flow: (5.000)#Flow rate {ml/min}, (Pre column pressure)
  #Pressure control
  0.00   End_Block
0.00   Phase: Equilibration
  0.00   Base: SameAsMain
  0.00   Inlet B: (B1)#Inlet B
  0.00   Inlet A: (A1)#Inlet A
  0.00   Gradient: (0.0)#Percent B (Equilibration) {%B}, 0.00 {base}
  0.00   System flow: (5.000)#Flow rate {ml/min}, (Pre column pressure)
  #Pressure control
  0.00   System wash: (15)#Fill system (Equilibration) {ml}, Injection
  valve (W1)
  0.00   Block: Hold until UV stable signal
    0.00   Base: Time, Any
    0.00   Watch UV parameters: 0.00 {mAU}, 1.00 {mAU}
    0.00   Hold until: UV, Stable signal, 0.03 {min}, 5.00 {base}
    0.00   End_Block
  0.00   Auto zero UV
  0.00   Block: Conditional equilibration
    0.00   Base: SameAsMain
    0.00   Block: Delay before equilibration watch
      0.00   Base: Volume, ColumnSameAsMain
      0.70   End_Block
    0.00   Watch UV parameters: 0.00 {mAU}, (0.10)#Accepted UV fluctuation
    (Equilibration) {mAU}
```

0.00 Watch: UV, Stable signal, (5.00)#Signal stable time (Equilibration) {min}, End block (10.00)#Max equilibration volume End_Block
0.00 Watch off: UV
0.00 Auto zero UV
0.00 End_Block
0.00 Phase: Sample Application
0.00 Base: SameAsMain
0.00 Block: Prepare sample loading
0.00 Base: SameAsMain
0.00 Set mark: ()#Sample_ID
0.00 System flow: 0.000 {ml/min}, Off
0.00 Sample pump wash: (Buffer)#Wash sample pump before sample application
0.00 End_Block
0.00 Block: Start frac (Sample Appl)
0.00 Base: SameAsMain
0.00 System flow: (3.000)#Flow rate (Sample Appl) {ml/min}, (Pre column pressure)#Pressure control
0.00 Fractionation in outlet valve: (Outlet 1)#Outlet frac start position (Sample Appl), (1)#Outlet frac max no of frac (Sample Appl), (20000.00)#Outlet frac volume (Sample Appl) {ml}
0.00 System flow: 0.000 {ml/min}, Off
0.00 End_Block
0.00 Block: Inject all sample
0.00 Base: Volume, ColumnSameAsMain
0.00 Alarm sample inlet air sensor: Disabled
0.00 Injection valve: Direct inject
0.00 Sample inlet: (S1)#Sample inlet
0.00 Sample flow: (3.000)#Flow rate (sample pump) {ml/min}, (Pre column pressure)#Pressure control (sample pump)
0.00 Hold until: Air sample inlet, Equals, Air, (Infinite)#Max sample volume {base}
0.00 End_Block
0.00 Block: Finalize sample injection
0.00 Base: Volume, ColumnSameAsMain
0.00 Sample inlet: Buffer
0.00 Sample flow: (1.000)#Reduced flow rate during sample injection {ml/min}, (Pre column pressure)#Pressure control (sample pump) (1.00)#Finalize injection volume End_Block
0.00 Sample flow: 0.000 {ml/min}, Off
0.00 Injection valve: Manual load
0.00 System flow: (3.000)#Flow rate (Sample Appl) {ml/min}, (Pre column pressure)#Pressure control
0.00 Block: Stop frac (Sample Appl)

```
      0.00   Base: SameAsMain
      0.00   Stop frac in outlet valve
      0.00   End_Block
    0.00   Sample pump wash: (Buffer)#Wash sample pump after sample
    application
    0.00   Alarm sample inlet air sensor: (Enabled)#Air sensor alarm on
    sample pump
    0.00   End_Block
  0.00   Phase: Column Wash
    0.00   Base: SameAsMain
    0.00   Inlet A: (A1)#Inlet A
    0.00   Inlet B: (B1)#Inlet B
    0.00   Gradient: (0.0)#Percent B (Column Wash) {%B}, 0.00 {base}
    0.00   System flow: (5.000)#Flow rate {ml/min}, (Pre column pressure)
    #Pressure control
    0.00   System wash: (15)#Fill system (Column Wash) {ml}, Injection valve
    (W1)
    0.00   Block: Start frac (Column Wash)
      0.00   Base: SameAsMain
      0.00   Fractionation in outlet valve: (Outlet 2)#Outlet frac start
      position (Column Wash), (1)#Outlet frac max no of frac (Column Wash),
      (20000.00)#Outlet frac volume (Column Wash) {ml}
      0.00   End_Block
    0.00   Block: Wash
      0.00   Base: SameAsMain
      (10.00)#Column wash volume   End_Block
    0.00   Block: Stop frac (Column Wash)
      0.00   Base: SameAsMain
      0.00   Stop frac in outlet valve
      0.00   End_Block
    0.00   End_Block
  0.00   Phase: Elution
    0.00   Base: SameAsMain
    0.00   Inlet A: (A1)#Inlet A
    0.00   Inlet B: (B1)#Inlet B
    0.00   Gradient: (26.5)#Percent B (Elution) {%B}, 0.00 {base}
    0.00   System flow: (1.000)#Flow rate (Elution) {ml/min}, (Pre column
    pressure)#Pressure control
    0.00   System wash: (15)#Fill system (Elution) {ml}, Injection valve (W1)
    0.00   Block: Start frac (Elution)
      0.00   Base: SameAsMain
      0.00   Last tube filled: (Pause)#Last tube filled action (Elution)
      0.00   Fractionation: Volume, (96 deep well plate)#Frac tube type
      (Elution), (0.50)#Frac volume (Elution) {ml}, (Next tube)#Frac start
      position (Elution)
```

```
      0.00   End_Block
    0.00   Block: Isocratic
      0.00   Base: SameAsMain
      (5.00)#Isocratic elution length   End_Block
    0.00   Block: Stop frac (Elution)
      0.00   Base: SameAsMain
      0.00   Stop fractionation
      0.00   End_Block
    0.00   End_Block
  0.00   Phase: Column Wash
    0.00   Base: SameAsMain
    0.00   Inlet A: (A1)#Inlet A
    0.00   Inlet B: (B1)#Inlet B
    0.00   Gradient: (100.0)#Percent B (Column Wash)_1 {%B}, 0.00 {base}
    0.00   System flow: (5.000)#Flow rate {ml/min}, (Pre column pressure)
    #Pressure control
    0.00   Block: Wash_1
      0.00   Base: SameAsMain
      (3.00)#Column wash volume_1   End_Block
    0.00   End_Block
  0.00   Phase: Column Wash
    0.00   Base: SameAsMain
    0.00   Inlet A: (A1)#Inlet A
    0.00   Inlet B: (B1)#Inlet B
    0.00   Gradient: (0.0)#Percent B (Column Wash)_2 {%B}, 0.00 {base}
    0.00   System flow: (5.000)#Flow rate {ml/min}, (Pre column pressure)
    #Pressure control
    0.00   Block: Wash_2
      0.00   Base: SameAsMain
      (3.00)#Column wash volume_2   End_Block
    0.00   End_Block
  0.00   Phase: Column Wash
    0.00   Base: SameAsMain
    0.00   Inlet A: (A1)#Inlet A
    0.00   Inlet B: (B1)#Inlet B
    0.00   Gradient: (100.0)#Percent B (Column Wash)_3 {%B}, 0.00 {base}
    0.00   System flow: (5.000)#Flow rate {ml/min}, (Pre column pressure)
    #Pressure control
    0.00   Block: Wash_3
      0.00   Base: SameAsMain
      (3.00)#Column wash volume_3   End_Block
    0.00   End_Block
  0.00   Phase: Column Wash
    0.00   Base: SameAsMain
```

0.00 Inlet A: (A1)#Inlet A
0.00 Inlet B: (B1)#Inlet B
0.00 Gradient: (0.0)#Percent B (Column Wash)_4 {%B}, 0.00 {base}
0.00 System flow: (5.000)#Flow rate {ml/min}, (Pre column pressure) #Pressure control
0.00 Block: Wash_4
0.00 Base: SameAsMain
(3.00)#Column wash volume_4 End_Block
0.00 End_Block

Method duration

Base:Time
Unit:min

Main method:

0.00 Base: CV, Vc=5.027 {ml}, (HisTrap HP, 5 ml)#Column type
0.00 Phase: Method Settings
0.00 Base: SameAsMain
0.00 Alarm pre column pressure: Enabled, (0.50)#Pre column pressure limit {MPa}, 0.00 {MPa}
0.00 Alarm delta column pressure: Enabled, (0.30)#Delta column pressure limit {MPa}, 0.00 {MPa}
0.00 Noise reduction UV: (2.5)#UV averaging time {sec}
0.00 Injection valve: Manual load
0.00 Column position: (1)#Column position, Down flow
0.00 pH valve: (In-line)#pH cell, (In-line)#Flow restrictor
0.00 Inlet A: (A1)#Inlet A
0.00 Inlet B: (B1)#Inlet B
0.00 Alarm inlet A air sensor: Enabled
0.00 Alarm sample inlet air sensor: (Enabled)#Air sensor alarm on sample pump
0.00 System flow: (5.000)#Flow rate {ml/min}, (Pre column pressure) #Pressure control
0.00 End_Block
0.00 Phase: Equilibration
0.00 Base: SameAsMain
0.00 Inlet B: (B1)#Inlet B
0.00 Inlet A: (A1)#Inlet A
0.00 Gradient: (0.0)#Percent B (Equilibration) {%B}, 0.00 {base}
0.00 System flow: (5.000)#Flow rate {ml/min}, (Pre column pressure) #Pressure control
0.00 System wash: (15)#Fill system (Equilibration) {ml}, Injection valve (W1)
0.00 Block: Hold until UV stable signal

```
      0.00   Base: Time, Any
      0.00   Watch UV parameters: 0.00 {mAU}, 1.00 {mAU}
      0.00   Hold until: UV, Stable signal, 0.03 {min}, 5.00 {base}
      0.00   End_Block
    0.00   Auto zero UV
    0.00   Block: Conditional equilibration
      0.00   Base: SameAsMain
      0.00   Block: Delay before equilibration watch
        0.00   Base: Volume, ColumnSameAsMain
        0.14   End_Block
      0.14   Watch UV parameters: 0.00 {mAU}, (0.10)#Accepted UV fluctuation
      (Equilibration) {mAU}
      0.14   Watch: UV, Stable signal, (5.00)#Signal stable time
      (Equilibration) {min}, End block
      (10.19)#Max equilibration volume   End_Block
    10.19   Watch off: UV
    10.19   Auto zero UV
    10.19   End_Block
  10.19   Phase: Sample Application
    10.19   Base: SameAsMain
    10.19   Block: Prepare sample loading
      10.19   Base: SameAsMain
      10.19   Set mark: ()#Sample_ID
      10.19   System flow: 0.000 {ml/min}, Off
      10.19   Sample pump wash: (Buffer)#Wash sample pump before sample
      application
      10.19   End_Block
    10.19   Block: Start frac (Sample Appl)
      10.19   Base: SameAsMain
      10.19   System flow: (3.000)#Flow rate (Sample Appl) {ml/min}, (Pre
      column pressure)#Pressure control
      10.19   Fractionation in outlet valve: (Outlet 1)#Outlet frac start
      position (Sample Appl), (1)#Outlet frac max no of frac (Sample Appl),
      (20000.00)#Outlet frac volume (Sample Appl) {ml}
      10.19   System flow: 0.000 {ml/min}, Off
      10.19   End_Block
    10.19   Block: Inject all sample
      10.19   Base: Volume, ColumnSameAsMain
      10.19   Alarm sample inlet air sensor: Disabled
      10.19   Injection valve: Direct inject
      10.19   Sample inlet: (S1)#Sample inlet
      10.19   Sample flow: (3.000)#Flow rate (sample pump) {ml/min}, (Pre
      column pressure)#Pressure control (sample pump)
      10.19   Hold until: Air sample inlet, Equals, Air, (Infinite)#Max
      sample volume {base}
```

```
      10.19   End_Block
    10.19   Block: Finalize sample injection
      10.19   Base: Volume, ColumnSameAsMain
      10.19   Sample inlet: Buffer
      10.19   Sample flow: (1.000)#Reduced flow rate during sample injection
      {ml/min}, (Pre column pressure)#Pressure control (sample pump)
      (11.19)#Finalize injection volume   End_Block
    11.19   Sample flow: 0.000 {ml/min}, Off
    11.19   Injection valve: Manual load
    11.19   System flow: (3.000)#Flow rate (Sample Appl) {ml/min}, (Pre
    column pressure)#Pressure control
    11.19   Block: Stop frac (Sample Appl)
      11.19   Base: SameAsMain
      11.19   Stop frac in outlet valve
      11.19   End_Block
    11.19   Sample pump wash: (Buffer)#Wash sample pump after sample
    application
    11.19   Alarm sample inlet air sensor: (Enabled)#Air sensor alarm on
    sample pump
    11.19   End_Block
  11.19   Phase: Column Wash
    11.19   Base: SameAsMain
    11.19   Inlet A: (A1)#Inlet A
    11.19   Inlet B: (B1)#Inlet B
    11.19   Gradient: (0.0)#Percent B (Column Wash) {%B}, 0.00 {base}
    11.19   System flow: (5.000)#Flow rate {ml/min}, (Pre column pressure)
    #Pressure control
    11.19   System wash: (15)#Fill system (Column Wash) {ml}, Injection valve
    (W1)
    11.19   Block: Start frac (Column Wash)
      11.19   Base: SameAsMain
      11.19   Fractionation in outlet valve: (Outlet 2)#Outlet frac start
      position (Column Wash), (1)#Outlet frac max no of frac (Column Wash),
      (20000.00)#Outlet frac volume (Column Wash) {ml}
      11.19   End_Block
    11.19   Block: Wash
      11.19   Base: SameAsMain
      (21.25)#Column wash volume   End_Block
    21.25   Block: Stop frac (Column Wash)
      21.25   Base: SameAsMain
      21.25   Stop frac in outlet valve
      21.25   End_Block
    21.25   End_Block
  21.25   Phase: Elution
    21.25   Base: SameAsMain
```

```
    21.25   Inlet A: (A1)#Inlet A
    21.25   Inlet B: (B1)#Inlet B
    21.25   Gradient: (26.5)#Percent B (Elution) {%B}, 0.00 {base}
    21.25   System flow: (1.000)#Flow rate (Elution) {ml/min}, (Pre column
    pressure)#Pressure control
    21.25   System wash: (15)#Fill system (Elution) {ml}, Injection valve
    (W1)
    21.25   Block: Start frac (Elution)
      21.25   Base: SameAsMain
      21.25   Last tube filled: (Pause)#Last tube filled action (Elution)
      21.25   Fractionation: Volume, (96 deep well plate)#Frac tube type
      (Elution), (0.50)#Frac volume (Elution) {ml}, (Next tube)#Frac start
      position (Elution)
      21.25   End_Block
    21.25   Block: Isocratic
      21.25   Base: SameAsMain
      (46.38)#Isocratic elution length   End_Block
    46.38   Block: Stop frac (Elution)
      46.38   Base: SameAsMain
      46.38   Stop fractionation
      46.38   End_Block
    46.38   End_Block
  46.38   Phase: Column Wash
    46.38   Base: SameAsMain
    46.38   Inlet A: (A1)#Inlet A
    46.38   Inlet B: (B1)#Inlet B
    46.38   Gradient: (100.0)#Percent B (Column Wash)_1 {%B}, 0.00 {base}
    46.38   System flow: (5.000)#Flow rate {ml/min}, (Pre column pressure)
    #Pressure control
    46.38   Block: Wash_1
      46.38   Base: SameAsMain
      (49.40)#Column wash volume_1   End_Block
    49.40   End_Block
  49.40   Phase: Column Wash
    49.40   Base: SameAsMain
    49.40   Inlet A: (A1)#Inlet A
    49.40   Inlet B: (B1)#Inlet B
    49.40   Gradient: (0.0)#Percent B (Column Wash)_2 {%B}, 0.00 {base}
    49.40   System flow: (5.000)#Flow rate {ml/min}, (Pre column pressure)
    #Pressure control
    49.40   Block: Wash_2
      49.40   Base: SameAsMain
      (52.42)#Column wash volume_2   End_Block
    52.42   End_Block
  52.42   Phase: Column Wash
```

```
    52.42   Base: SameAsMain
    52.42   Inlet A: (A1)#Inlet A
    52.42   Inlet B: (B1)#Inlet B
    52.42   Gradient: (100.0)#Percent B (Column Wash)_3 {%B}, 0.00 {base}
    52.42   System flow: (5.000)#Flow rate {ml/min}, (Pre column pressure)
    #Pressure control
    52.42   Block: Wash_3
      52.42   Base: SameAsMain
      (55.43)#Column wash volume_3   End_Block
    55.43   End_Block
  55.43   Phase: Column Wash
    55.43   Base: SameAsMain
    55.43   Inlet A: (A1)#Inlet A
    55.43   Inlet B: (B1)#Inlet B
    55.43   Gradient: (0.0)#Percent B (Column Wash)_4 {%B}, 0.00 {base}
    55.43   System flow: (5.000)#Flow rate {ml/min}, (Pre column pressure)
    #Pressure control
    55.43   Block: Wash_4
      55.43   Base: SameAsMain
      (58.45)#Column wash volume_4   End_Block
    58.45   End_Block
```

Total Volume (approximately):187.70 ml
Total Time (approximately):58.45 min

Variable list

Phase	Block	Variable	Value	Unit
Method Settings	Main	Column type	HisTrap HP, 5 ml	
Method Settings	METHOD SETTINGS	Pre column pressure limit	0.50	{MPa}
		Delta column pressure limit	0.30	{MPa}
		UV averaging time	2.5	{sec}
		Column position	1	
		pH cell	In-line	
		Flow restrictor	In-line	
Method Settings,...	METHOD SETTINGS,...	Inlet A	A1	
		Inlet B	B1	

**Phase**	**Block**	**Variable**	**Value**	**Unit**
		Air sensor alarm on sample pump	Enabled	
		Flow rate	5.000	{ml/min}
		Pressure control	Pre column pressure	
Equilibration	EQUILIBRATION	Percent B (Equilibration)	0.0	{%B}
		Fill system (Equilibration)	15	{ml}
Equilibration	Conditional equilibration	Accepted UV fluctuation (Equilibration)	0.10	{mAU}
		Signal stable time (Equilibration)	5.00	{min}
		Max equilibration volume	10.00	{CV}
Sample Application	Prepare sample loading	Sample_ID		
		Wash sample pump before sample application	Buffer	
Sample Application	Start frac (Sample Appl),...	Flow rate (Sample Appl)	3.000	{ml/min}
Sample Application	Start frac (Sample Appl)	Outlet frac start position (Sample Appl)	Outlet 1	
		Outlet frac max no of frac (Sample Appl)	1	
		Outlet frac volume (Sample Appl)	20000.00	{ml}
Sample Application	Inject all sample	Sample inlet	S1	
		Flow rate (sample pump)	3.000	{ml/min}

**Phase**	**Block**	**Variable**	**Value**	**Unit**
Sample Application	Inject all sample,...	Pressure control (sample pump)	Pre column pressure	
Sample Application	Inject all sample	Max sample volume	Infinite	{base}
Sample Application	Finalize sample injection	Reduced flow rate during sample injection	1.000	{ml/min}
		Finalize injection volume	1.00	{ml}
Sample Application	SAMPLE APPLICATION	Wash sample pump after sample application	Buffer	
Column Wash	COLUMN WASH	Percent B (Column Wash)	0.0	{%B}
		Fill system (Column Wash)	15	{ml}
Column Wash	Start frac (Column Wash)	Outlet frac start position (Column Wash)	Outlet 2	
		Outlet frac max no of frac (Column Wash)	1	
		Outlet frac volume (Column Wash)	20000.00	{ml}
Column Wash	Wash	Column wash volume	10.00	{CV}
Elution	ELUTION	Percent B (Elution)	26.5	{%B}
		Flow rate (Elution)	1.000	{ml/min}
		Fill system (Elution)	15	{ml}
Elution	Start frac (Elution)	Last tube filled action (Elution)	Pause	
		Frac tube type (Elution)	96 deep well plate	
		Frac volume (Elution)	0.50	{ml}

Phase	Block	Variable	Value	Unit
		Frac start position (Elution)	Next tube	
Elution	Isocratic	Isocratic elution length	5.00	{CV}
Column Wash	COLUMN WASH_1	Percent B (Column Wash)_1	100.0	{%B}
Column Wash	Wash_1	Column wash volume_1	3.00	{CV}
Column Wash	COLUMN WASH_2	Percent B (Column Wash)_2	0.0	{%B}
Column Wash	Wash_2	Column wash volume_2	3.00	{CV}
Column Wash	COLUMN WASH_3	Percent B (Column Wash)_3	100.0	{%B}
Column Wash	Wash_3	Column wash volume_3	3.00	{CV}
Column Wash	COLUMN WASH_4	Percent B (Column Wash)_4	0.0	{%B}
Column Wash	Wash_4	Column wash volume_4	3.00	{CV}

Method information

Properties:

Method name: C6 Nickel affinity With Equilibration With Quick Wash and 1 M Imidazole
Method created by: Default
Date of creation: 3/17/2022 8:24:23 AM -04:00
Created for system: Hardy
Date of last modification: 5/6/2025 10:29:25 AM -04:00
Method last modified by: Default

Strategy name: AKTApure25_322

Start protocol

Method Items to display at method start:
Fraction Collector

Variable List
Text Instructions
Gradient
Columns
Result Name And Location

Result name and location

Result folder name: /DefaultHome
Result name: C6 Nickel affinity With Equilibration With Quick Wash and 1 M Imidazole (Type: Method name) without unique identifier

Columns

HisTrap HP, 5 ml
Run Parameters

Technique	Affinity	
Column volume	5.027	ml
Max pre-column pressure	0.50	MPa
Max delta column pressure	0.30	MPa
Default flow rate	5.00	ml/min
Max flow rate	20.00	ml/min
Default linear flow rate	149.21	cm/h
Max linear flow rate	596.83	cm/h
Min pH value (short term)	2	
Max pH value (short term)	14	
Min pH value (long term)	3	
Max pH value (long term)	13	

Details

Hardware diameter	1.60	cm
Bed height	2.50	cm
Typical loading range	1-150	mg
Total liquid volume (Vt)	4.27	ml
Void volume (Vo)		ml
Typical peak width at base	5.00	ml
Average particle diameter	34.00	µm
Molecular weight range		Mr

Ordering information

Name	HisTrap HP, 5 ml
Code number	17-5248-02
Resin name	Ni Sepharose High Performance
Resin code number	17-5268-02
Hardware name	HiTrap 5ml
Hardware code number	
