# Supplementary material for: An NMR sample preparation case study: Considerations for the self-destructive protease caspase-6
Source: PLoS One. 2025 Nov 21;20(11):e0337291. doi: 10.1371/journal.pone.0337291 (PMC12637907; doi:10.1371/journal.pone.0337291)
Supplement: Attachment — Submitted filename: Response to Reviewers.docx [file pone.0337291.s011.docx]

Sept 24, 2025

Dear Dr. Ajit Prakash,

We are pleased to submit a revised manuscript entitled “An NMR Sample Preparation Case Study: Considerations for the Self-Destructive Protease Caspase-6**”** PONE-D-25-34737 as a research article for *PLOS ONE*.  We appreciate the reviewers’ comments and feedback. Many of the comments were valuable in improving clarification and readability. We conducted time-dependent stability testing by NMR and included that data as Supplementary Figure 2. We also have included the requested supplementary materials such as NMR sample and spectrum information (S3 Table) as well as uncropped and unaltered SDS-PAGE gels (S1_raw_images). All the changes requested in the main text of the manuscript are detailed in this “Response to Reviewers” document as well as in the “Revised Manuscript with Track Changes” document. We hope that these substantive changes will enable acceptance of this revised manuscript by *PLOS ONE*.

Very truly yours,

Jeanne Hardy

-----------------------------------------------------------------------------------------------------------------------------------------

The following is in response to comments received from reviewers on our manuscript “An NMR Sample Preparation Case Study: Considerations for the Self-Destructive Protease Caspase-6.” All responses are shown in blue text.

1. Please ensure that your manuscript meets PLOS ONE's style requirements, including those for file naming. The PLOS ONE style templates can be found at

<https://journals.plos.org/plosone/s/file?id=wjVg/PLOSOne_formatting_sample_main_body.pdf>

and

[https://journals.plos.org/plosone/s/file?id=ba62/PLOSOne_formatting_sample_title_authors_affiliations.pdf](https://nam10.safelinks.protection.outlook.com/?url=https%3A%2F%2Ftrack.editorialmanager.com%2FCL0%2Fhttps%3A%252F%252Fjournals.plos.org%252Fplosone%252Fs%252Ffile%253Fid%3Dba62%252FPLOSOne_formatting_sample_title_authors_affiliations.pdf%2F1%2F010f0198ae05abb6-6ce8af03-d2b9-4170-9a75-cce2059db3ae-000000%2FF7pXYTIWjvG2F7PtL5ke0r25v3q2ZMRRfgCHOa6EFuk%3D224&data=05%7C02%7Cnkuzio%40umass.edu%7Ce48dc1c2c63545461b4d08dddc0723b1%7C7bd08b0b33954dc194bbd0b2e56a497f%7C0%7C0%7C638908645655639935%7CUnknown%7CTWFpbGZsb3d8eyJFbXB0eU1hcGkiOnRydWUsIlYiOiIwLjAuMDAwMCIsIlAiOiJXaW4zMiIsIkFOIjoiTWFpbCIsIldUIjoyfQ%3D%3D%7C0%7C%7C%7C&sdata=6n%2FhEZ3tS3sU%2BBEJvN600dTjxdr37M4eHznycuM%2B1CI%3D&reserved=0)

2. We note that this submission includes NMR spectroscopy data. We would recommend that you include the following information in your methods section or as Supporting Information files:
1) The make/source of the NMR instrument used in your study, as well as the magnetic field strength. For each individual experiment, please also list: the nucleus being measured; the sample concentration; the solvent in which the sample is dissolved and if solvent signal suppression was used; the reference standard and the temperature.

This comment was addressed by the addition of S3 Table which outlines samples and parameters for all presented NMR experiments, clarification of reference to S3 Table in the NMR Data Collection methods section, and the addition of S3 Table legend at the end of the “Revised Manuscript with Track Changes” document.

2) A list of the chemical shifts for all compounds characterised by NMR spectroscopy, specifying, where relevant: the chemical shift (δ), the multiplicity and the coupling constants (in Hz), for the appropriate nuclei used for assignment.

This comment was addressed by the addition of a line at the end of Fig. 7 legend indicating where the assignments of the ^13^C-Ileδ1-methyl labeled casp-6 D179 CT [^1^H,^13^C] HMQC spectrum can be assessed in the biological magnetic resonance bank. No small molecules were assigned in this study. “NMR assignments for the ^13^C-Ileδ1-methyl labeled casp-6 D179 CT ^1^H-^13^C HMQC spectrum can now be found at BMRB entry 53341 in the biological magnetic resonance bank.”

3) The full integrated NMR spectrum, clearly labelled with the compound name and chemical structure.

No small molecules were characterized by NMR in this study, so no integrated NMR spectra were included.

We also strongly encourage authors to provide primary NMR data files, in particular for new compounds which have not been characterised in the existing literature. Authors should provide the acquisition data, FID files and processing parameters for each experiment, clearly labelled with the compound name and identifier, as well as a structure file for each provided dataset. See our list of recommended repositories here: [https://journals.plos.org/plosone/s/recommended-repositories](https://nam10.safelinks.protection.outlook.com/?url=https%3A%2F%2Ftrack.editorialmanager.com%2FCL0%2Fhttps%3A%252F%252Fjournals.plos.org%252Fplosone%252Fs%252Frecommended-repositories%2F1%2F010f0198ae05abb6-6ce8af03-d2b9-4170-9a75-cce2059db3ae-000000%2F9ioelrv1Z0_5Gr_CV4oxctpPftfusQMnggECY6MalOI%3D224&data=05%7C02%7Cnkuzio%40umass.edu%7Ce48dc1c2c63545461b4d08dddc0723b1%7C7bd08b0b33954dc194bbd0b2e56a497f%7C0%7C0%7C638908645655657132%7CUnknown%7CTWFpbGZsb3d8eyJFbXB0eU1hcGkiOnRydWUsIlYiOiIwLjAuMDAwMCIsIlAiOiJXaW4zMiIsIkFOIjoiTWFpbCIsIldUIjoyfQ%3D%3D%7C0%7C%7C%7C&sdata=nwohAUefKXPlwGx4aaanKL3Q5X3NaoAGhZdi2sXyf5w%3D&reserved=0).

This comment was addressed by the addition of a line at the end of Fig. 7 legend indicating where the assignments of the ^13^C-Ileδ1-methyl labeled casp-6 D179 CT [^1^H,^13^C] HMQC spectrum can assessed in the biological magnetic resonance bank and the presence of S1 Data which includes all NMR spectrum files for 1D ^1^H experiments.

3. PLOS ONE now requires that authors provide the original uncropped and unadjusted images underlying all blot or gel results reported in a submission’s figures or Supporting Information files. This policy and the journal’s other requirements for blot/gel reporting and figure preparation are described in detail at <https://journals.plos.org/plosone/s/figures#loc-blot-and-gel-reporting-requirements> and <https://journals.plos.org/plosone/s/figures#loc-preparing-figures-from-image-files>. When you submit your revised manuscript, please ensure that your figures adhere fully to these guidelines and provide the original underlying images for all blot or gel data reported in your submission. See the following link for instructions on providing the original image data: <https://journals.plos.org/plosone/s/figures#loc-original-images-for-blots-and-gels>.

This comment was addressed by the addition of a supporting information file titled "S1_raw_images.pdf” that contains all the original uncropped SDS-PAGE gel images. All of the images have been annotated according to PLOS ONE requirements.

In your cover letter, please note whether your blot/gel image data are in Supporting Information or posted at a public data repository, provide the repository URL if relevant, and provide specific details as to which raw blot/gel images, if any, are not available. Email us at [plosone@plos.org](mailto:plosone@plos.org) if you have any questions.

4. If the reviewer comments include a recommendation to cite specific previously published works, please review and evaluate these publications to determine whether they are relevant and should be cited. There is no requirement to cite these works unless the editor has indicated otherwise.

   [Note: HTML markup is below. Please do not edit.]

   Reviewers' comments:

   Reviewer's Responses to Questions

**Comments to the Author (Reviewer #1)**

1. Is the manuscript technically sound, and do the data support the conclusions?

The manuscript must describe a technically sound piece of scientific research with data that supports the conclusions. Experiments must have been conducted rigorously, with appropriate controls, replication, and sample sizes. The conclusions must be drawn appropriately based on the data presented.

Reviewer #1: Yes

Reviewer #2: Yes

2. Has the statistical analysis been performed appropriately and rigorously?

Reviewer #1: Yes

Reviewer #2: Yes

3. Have the authors made all data underlying the findings in their manuscript fully available?

The [PLOS Data policy](https://nam10.safelinks.protection.outlook.com/?url=http%3A%2F%2Ftrack.editorialmanager.com%2FCL0%2Fhttp%3A%252F%252Fwww.plosone.org%252Fstatic%252Fpolicies.action%2523sharing%2F1%2F010f0198ae05abb6-6ce8af03-d2b9-4170-9a75-cce2059db3ae-000000%2FCDN64-o43a95IvA-Yox9SgO8ISogOk6uBwThUJPq_M4%3D224&data=05%7C02%7Cnkuzio%40umass.edu%7Ce48dc1c2c63545461b4d08dddc0723b1%7C7bd08b0b33954dc194bbd0b2e56a497f%7C0%7C0%7C638908645655672213%7CUnknown%7CTWFpbGZsb3d8eyJFbXB0eU1hcGkiOnRydWUsIlYiOiIwLjAuMDAwMCIsIlAiOiJXaW4zMiIsIkFOIjoiTWFpbCIsIldUIjoyfQ%3D%3D%7C0%7C%7C%7C&sdata=1maWwEr6bJYNU1uxtpWY3ny4qGZuqJX3OEz2FCk9rDo%3D&reserved=0) requires authors to make all data underlying the findings described in their manuscript fully available without restriction, with rare exception (please refer to the Data Availability Statement in the manuscript PDF file). The data should be provided as part of the manuscript or its supporting information, or deposited to a public repository. For example, in addition to summary statistics, the data points behind means, medians and variance measures should be available. If there are restrictions on publicly sharing data—e.g. participant privacy or use of data from a third party—those must be specified.

Reviewer #1: Yes

Reviewer #2: Yes

4. Is the manuscript presented in an intelligible fashion and written in standard English?

PLOS ONE does not copyedit accepted manuscripts, so the language in submitted articles must be clear, correct, and unambiguous. Any typographical or grammatical errors should be corrected at revision, so please note any specific errors here.

Reviewer #1: Yes

Reviewer #2: Yes

5. Review Comments to the Author

Please use the space provided to explain your answers to the questions above. You may also include additional comments for the author, including concerns about dual publication, research ethics, or publication ethics. (Please upload your review as an attachment if it exceeds 20,000 characters)

Reviewer #1: Summary:
This manuscript describes the successful optimization of an NMR sample preparation protocol for Caspase-6, a self-degrading protease that has been historically difficult to study using solution NMR. The authors systematically test different growth, purification, and buffer exchange conditions to obtain stable, concentrated, isotopically labeled samples. Their optimized protocol improves yield and spectral quality by eliminating steps that cause protein loss, such as anion exchange and spin filtration. The study also evaluates multiple constructs and conditions to find one that is NMR-compatible. This work offers practical guidance for other researchers working on similarly unstable or low-yield proteins.

Strengths:
1. The study addresses a longstanding technical challenge in the preparation of an NMR-compatible form of Caspase-6, a self-cleaving and aggregation-prone protease.
2. The experimental design is thorough and data-driven leading to a final protocol that is simple, reproducible, and avoids problematic steps like anion exchange and centrifugal concentration.
3. The authors choose an appropriate construct (D179CT) and NMR approach (Ileδ1 13CH3 methyl HMQC) for a first step in structural work.
4. The manuscript is well organized, and the conclusions are well supported by experimental outcomes.
5. The work is broadly applicable to researchers preparing dynamic or unstable proteins for biophysical characterization.


Major Comments:
1. The manuscript states that Ni-affinity purification alone provided sufficient purity and improved yield. However, this claim would be stronger if the authors included direct comparisons of yield, concentration, and purity between samples that did and did not undergo anion exchange. An SDS-PAGE image or protein quantification comparing the two workflows would be useful.

We thank the reviewer for this suggestion to make the claim stronger. This comment was addressed by the addition of two panels to figure 3 showing a nickel-affinity purification gel (panel D) and PD-10 buffer exchange gel (panel E). These serve as a direct comparison to the nickel-affinity and anion exchange purification gel found in panel C. Additionally, explanation was added to the corresponding text on page 10 and figure 3 legend supporting this claim. “This was supported by the adequate purity of the nickel elution fractions (“Ni Elu”, Fig. 3C) and lack of contaminant protein removed during the anion-exchange steps (“QFT” and “Q Wash”, Fig. 3C). Additionally, using solely a Ni-affinity purification (**Fig. 3D**) followed by buffer exchange with a PD-10 desalting column (**Fig. 3E**) resulted in an increased growth and purification yield of ~1 mg/L compared to ~0.5 mg/L that were obtained from a Ni-affinity and anion-exchange purification.”

2. The authors should clarify in the Introduction or early Results that spin filters caused significant sample loss and were avoided throughout the workflow. Including specific protein recovery data comparing desalting columns to centrifugal filters will support this decision and strengthen the general takeaway.

We thank the reviewer for this suggestion; this comment was addressed by the addition of a sentence on page 4 in the introduction explicitly stating the inefficiency of using spin filters for concentration and buffer exchange. “In particular, common concentration and buffer exchange methods using spin filters have been unreliable as this often leads to percent recoveries of <10% for casp-6, especially when working with concentrations below 40 µM.”

3. The various optimizations are scattered throughout the text. A summary comparing the initial and final workflows with numerical data for yield (mg/L), concentration (µM), recovery %, and NMR suitability would help readers quickly understand the benefits of each change.

We thank the reviewer for pointing out that this can be difficult to follow. This comment was addressed by the addition of explicit comparison of numerical data before and after optimization including:

on page 6 “Interestingly, protein yield was approximately 5 mg/L on average for cultures grown in M9 or 2x M9 minimal media, which was significantly higher than 1 mg/L which was observed for those grown in 2xYT, a rich media (Fig. 2B).”

on page 8 “Combining the improved step elution profile (230 mM NaCl), this lower flow rate (1 mL/min) and smaller fraction volumes (0.5 mL) permitted selection of more concentrated samples (Fig. 3B) of ~100 µM instead of 40 µM which is achieved with the traditional elution protocol.”

on page 10 “Additionally, using solely a Ni-affinity purification (Fig. 3D) followed by buffer exchange with a PD-10 desalting column (Fig. 3E) resulted in an increased growth and purification yield of ~1 mg/L compared to ~0.5 mg/L that were obtained from a Ni-affinity and anion-exchange purification.”

and on page 12 “This allowed for efficient buffer exchange with up to 70% recovery, a dramatic improvement from <10% which is seen with traditional centrifugal spin filter methods”.

4. The authors should report how many Ileδ1 methyl peaks were observed in the HMQC spectrum relative to the expected number based on sequence. This will help assess how well-folded and NMR-ready the sample is.

This comment was addressed by the addition of two sentences in the conclusions on page 19 that discuss the observed vs expected number of Ile peaks in the ^1^H-^13^C HMQC spectrum. “Given that we are particularly interested in a structural interconversion occurring in the protein core, we decided to focus our attention on the ^1^H-^13^C HMQC spectrum of the 12 Ileδ1-^13^CH_3_ groups of casp-6 D179 CT (Fig. 7). The ^1^H-^13^C HMQC spectrum of Ileδ1-^13^CH_3_-labeled casp-6 showed 13 isoleucine peaks, which was expected as some Ile residues are known to undergo conformational exchange.”

5. The authors should comment on signal-to-noise ratio, chemical shift dispersion, and linewidth in the methyl HMQC spectrum. Even if qualitative, this information will support claims of spectral quality and sample integrity.

This comment was addressed by the addition of a sentence in the conclusions on page 19 that discusses the optimal spectral characteristics of the ^1^H-^13^C HMQC spectrum consistent with a properly folded and stable protein. “Additionally, modest signal-to-noise, well-dispersed chemical shifts, and linewidth were consistent with a stable and well-folded, yet dynamic, protein suggesting successful sample optimization.”

6. The authors should assess how stable the Caspase-6 samples are over a 24- to 48- hour period at room temperature or 25°C. Overlays of spectra at different time points or a description of peak loss or aggregation would help others plan experiments using this protocol.

This comment was addressed by the addition of S2 Fig which shows stability tests that were performed on Ileδ1-^13^CH_3_-labeled casp-6 D179 CT. These spectra showed that under the optimized conditions casp-6 was stable for up to five days, emphasizing the potential for time-intensive NMR experiments. This was also referenced on page 15 with the addition of the sentence “Subsequent stability tests on methyl-labeled casp-6 further supported the optimization showing no significant reduction in peak intensity for up to five days when incubated at 25 °C (S2 Fig).”

7. If the authors have successfully expressed and collected a 1H-15N HSQC spectrum for the full-length C163S variant, it would be valuable to include this data, even if unassigned. This will strengthen the claim that the optimized protocol supports analysis of other constructs.

While we agree that this would be valuable insight into the applicability of these methods for other constructs, this is planned for future experiments and a ^1^H-^15^N HSQC has not yet been obtained for this construct.

8. The manuscript states that these findings may apply to other proteases. The authors should be more specific about which steps, such as choice of Tris pH 8.5, inclusion of DTT, use of glycerol, or buffer exchange strategy, are likely to benefit other unstable or aggregation-prone proteins.

We thank the reviewer for suggesting clarification on this point. This comment was addressed by addition of a sentence on page 19 that emphasizes the importance of these optimizations for difficult to concentrate proteins “Specifically, optimizing buffer conditions, as well as concentration and exchange methods for self-destructive proteins like proteases is critical for obtaining highly-concentrated yet stable samples.”

Minor Comments:
1. Standardize the use of “Caspase-6” or “Casp-6” throughout the manuscript for consistency.

We thank the reviewer for catching this inconsistency. This comment was addressed by changing occurrences of “caspase-6” to “casp-6” in the main text. “Caspase-6” was used in the abstract and the first time it was used in the main text.

2. Ensure that all figures (particularly Fig 2A, 2C, 3B, 4B, 6A-D) include properly labeled axes, units, and legends. Clarify what the error bars represent (SD or SEM), and indicate the number of replicates (n).

Thank you for this point. To clarify these issues, we have added clarifying statements in the legends of Fig 2, Fig 4 and Fig 5 describing the number of replicates, the symbols used, fraction numbers and error bar representation. We have added axis labels in Fig. 4B, 6A-D.

3. In the Methods section, clarify whether IPTG concentration, growth temperature, and induction timing were fixed based on prior studies or tested as part of this optimization.

This comment was addressed by the addition of a clarifying statement at the beginning of the methods section pertaining to the expression and labeling of caspase-6 on page 20. “Protein overexpression parameters that were not optimized in this study, including isopropyl β-D-1-thiogalactopyranoside (IPTG) concentration, growth temperature, induction time, and induction temperature were performed as previously described [16] or according to standard isotopic-labeling procedures.”

4. All supplemental materials (S1 Protocol, S1 Fig, S2 Table) should be cited at appropriate points in the main text where their content is relevant. Avoid listing them only in the Supporting Information section.

This comment was addressed by ensuring all supplementary information items are appropriately cited in the main text, S1 Table on page 5, S1 Fig on page 7, S1 Protocol on page 10, S2 Table of page 13, S2 Fig on page 15, and S3 Table on page 13. The file entitled “S1 Data,” which contains all the raw data associated with Figures 2-6, was not cited in the main text, but is noted in its own figure legend, as instructed.

5. In the abstract, revise the sentence “it remained impossible to analyze caspase-6 by NMR” to something more direct, such as “Prior attempts to analyze Caspase-6 by NMR failed due to challenges in sample preparation.”

We thank the reviewer for this excellent suggestion! This comment was addressed by modifying the corresponding sentence in the abstract to “Prior attempts to study caspase-6 by NMR have failed due to challenges in sample preparation and insufficient protein concentration.”

**Review Comments to the Author (Reviewer #2)**

Reviewer #2: The section is scientifically sound and well-documented, but it would benefit from:

Condensing repeated explanations,

Providing brief quantitative context when referencing figures,

Structuring into smaller paragraphs for readability,

Emphasizing the key conclusion more prominently.

6. PLOS authors have the option to publish the peer review history of their article ([what does this mean?](https://nam10.safelinks.protection.outlook.com/?url=https%3A%2F%2Ftrack.editorialmanager.com%2FCL0%2Fhttps%3A%252F%252Fjournals.plos.org%252Fplosone%252Fs%252Feditorial-and-peer-review-process%2523loc-peer-review-history%2F1%2F010f0198ae05abb6-6ce8af03-d2b9-4170-9a75-cce2059db3ae-000000%2FQgp5tWKRpAUrmzitOI1fRCLrIw-Na4dwVjCeVN7x3wY%3D224&data=05%7C02%7Cnkuzio%40umass.edu%7Ce48dc1c2c63545461b4d08dddc0723b1%7C7bd08b0b33954dc194bbd0b2e56a497f%7C0%7C0%7C638908645655686709%7CUnknown%7CTWFpbGZsb3d8eyJFbXB0eU1hcGkiOnRydWUsIlYiOiIwLjAuMDAwMCIsIlAiOiJXaW4zMiIsIkFOIjoiTWFpbCIsIldUIjoyfQ%3D%3D%7C0%7C%7C%7C&sdata=%2Btjzw09rPYS8CMCgETsXyJVNsqGm%2BOLbuMiTLq3Yik4%3D&reserved=0)). If published, this will include your full peer review and any attached files.


If you choose “no”, your identity will remain anonymous but your review may still be made public.


**Do you want your identity to be public for this peer review?** For information about this choice, including consent withdrawal, please see our [Privacy Policy](https://nam10.safelinks.protection.outlook.com/?url=https%3A%2F%2Ftrack.editorialmanager.com%2FCL0%2Fhttps%3A%252F%252Fwww.plos.org%252Fprivacy-policy%2F1%2F010f0198ae05abb6-6ce8af03-d2b9-4170-9a75-cce2059db3ae-000000%2F0eRzyh_FTHUsZKYnYd_R089uDQH_a8LBcT69PVVKNmU%3D224&data=05%7C02%7Cnkuzio%40umass.edu%7Ce48dc1c2c63545461b4d08dddc0723b1%7C7bd08b0b33954dc194bbd0b2e56a497f%7C0%7C0%7C638908645655700905%7CUnknown%7CTWFpbGZsb3d8eyJFbXB0eU1hcGkiOnRydWUsIlYiOiIwLjAuMDAwMCIsIlAiOiJXaW4zMiIsIkFOIjoiTWFpbCIsIldUIjoyfQ%3D%3D%7C0%7C%7C%7C&sdata=TPyQ1SIcJWnL%2FehU2D%2Fgi1wyNnEXAh3TNgAXD%2BVbt%2F8%3D&reserved=0).

Reviewer #1: No

Reviewer #2: **Yes:** DR SAI KUMAR BADAM

[NOTE: If reviewer comments were submitted as an attachment file, they will be attached to this email and accessible via the submission site. Please log into your account, locate the manuscript record, and check for the action link "View Attachments". If this link does not appear, there are no attachment files.]

While revising your submission, please upload your figure files to the Preflight Analysis and Conversion Engine (PACE) digital diagnostic tool, [https://pacev2.apexcovantage.com/](https://nam10.safelinks.protection.outlook.com/?url=https%3A%2F%2Ftrack.editorialmanager.com%2FCL0%2Fhttps%3A%252F%252Fpacev2.apexcovantage.com%252F%2F1%2F010f0198ae05abb6-6ce8af03-d2b9-4170-9a75-cce2059db3ae-000000%2FD6pXJe8lovWVhgbmSf39eY2DGvtV8piVqRvXfRGv9b4%3D224&data=05%7C02%7Cnkuzio%40umass.edu%7Ce48dc1c2c63545461b4d08dddc0723b1%7C7bd08b0b33954dc194bbd0b2e56a497f%7C0%7C0%7C638908645655715818%7CUnknown%7CTWFpbGZsb3d8eyJFbXB0eU1hcGkiOnRydWUsIlYiOiIwLjAuMDAwMCIsIlAiOiJXaW4zMiIsIkFOIjoiTWFpbCIsIldUIjoyfQ%3D%3D%7C0%7C%7C%7C&sdata=Xg1yh7tAMlnC1UzOgO5YGJIchZUez08JYKtPxPynqJo%3D&reserved=0). PACE helps ensure that figures meet PLOS requirements. To use PACE, you must first register as a user. Registration is free. Then, login and navigate to the UPLOAD tab, where you will find detailed instructions on how to use the tool. If you encounter any issues or have any questions when using PACE, please email PLOS at [figures@plos.org](mailto:figures@plos.org). Please note that Supporting Information files do not need this step.

**An NMR Sample Preparation Case Study: Considerations for the Self-Destructive Protease Paper Review and Comments by: Dr SAI KUMAR BADAM**

**1.** The introduction **is** scientifically solid and well-supported and the introduction does an excellent job of situating caspase-6 (casp-6) in the context of the broader caspase family, covering structural, functional, and pathological aspects.

**Results:**

**Caspase-6 can be satisfactorily overexpressed in *E. coli* in 2x M9 minimal media:**

**Strengths**

The section clearly moves from the problem (need for isotopically labeled casp-6) → method (growth in rich vs minimal media) → variable testing (M9 vs 2×M9, induction OD) → conclusion (optimal growth conditions).

Growth media composition, inoculation strategies, temperature, IPTG concentration, and OD600 ranges are clearly stated, making replication possible.

Mention of previous studies ([50, 51]) to justify testing 2×M9 provides good context for why this variable was examined.

**Areas for Improvement**

Several sentences repeat the same idea in slightly different forms (e.g., higher yield in minimal media is explained in multiple places).

We thank the reviewer for noting that this is confusing. This comment was addressed by clarification on page 6 stating that the first increase in protein yield that is being discussed is between M9 and 2x M9 minimal media “This has led to greater protein yields in 2x M9 compared to M9 minimal media as the *E. coli* remains more viable at a higher OD_600_”, while the second time this is discussed is between minimal media and nutrient-rich media (2xYT) “Interestingly, protein yield was approximately 5 mg/L on average for cultures grown in M9 or 2x M9 minimal media, which was significantly higher than 1 mg/L which was observed for those grown in 2xYT, a rich media (Fig. 2B).”

Figures (e.g., Fig. 2B, Fig. 2D) are referenced, but the reader must infer trends from the text. Briefly **state the key numeric differences or statistical significance** to make the results standalone from the figures.

We thank the reviewer for their suggestion to make this clearer. This comment was addressed by the addition of more specific values from Fig. 2 data to the main text on page 6 “Interestingly, protein yield was approximately 5 mg/L on average for cultures grown in M9 or 2x M9 minimal media, which was significantly higher than 1 mg/L which was observed for those grown in 2xYT, a rich media (Fig. 2B)” and on page 7 “For both M9 and 2x M9 media an induction OD_600_ of 1.1 yielded the greatest amount of protein produced per liter at 5-6 mg/L, with yield dropping to 3-4 mg/L as induction OD_600_ increased (Fig. 2D). Increasing induction OD_600_ also corresponded with an unexpected decrease in cell pellet size from 4 g/L to 3 g/L”. When applicable, specific significance of the results was also added.

The final sentence does give the optimal condition (2×M9, OD600 = 1.1), but this could be **highlighted more strongly** as the experimental takeaway.

Example:

Based on these findings, the optimal expression condition for isotopically labeled casp-6 in E. coli is growth in 2×M9 minimal media with induction at OD600 = 1.1 using 1 mM IPTG at 20 °C for 18 h.

We thank the reviewer for their comment. This comment was addressed by rephrasing the last two sentences of this section on page 7 to make it more clear and direct. “Despite no significant difference in the overall amount of protein that was expressed in M9 or 2x M9 minimal media with both compositions yielding approximately 5 mg/L (Fig. 2D), 2x M9 minimal media appeared more tolerant to induction at a higher OD_600_, likely due to the increased buffering capacity. Based on these data, the optimal conditions for casp-6 overexpression consisted of growth in 2x M9 minimal media to an OD_600_ of 1.1 followed by induction for 18 hours at 20 °C.”

**Elution concentration improved by optimizing flow rates and 150** **gradients during purification:**

**Strengths**

You start with a well-defined problem (sample loss during concentration when <40 µM) and link it directly to the need for optimizing purification parameters.

You reference your lab’s and others’ previous purification strategies, making it clear this is an incremental but meaningful improvement.

Flow rate, elution profile, and fraction volume are discussed in a sequence that mirrors how a reader might troubleshoot the same issue.

You explicitly tie Protocol 4’s conditions (230 mM NaCl step elution + 1 mL/min + 0.5 mL fractions) to the desired outcome (higher concentration and minimal loss during buffer exchange).

**Areas for Improvement**

The section is heavy on procedural parameters (flow rates, NaCl concentrations, column types) in one long paragraph. Splitting it into **two smaller paragraphs**—(1) rationale & design changes, (2) results & optimal conditions—would improve readability.

This comment was addressed by restructuring the corresponding section on pages 7 and 8 to discuss optimization rationale and design followed by results as suggested by Reviewer #2.

Some sentences feel more like *methods* than *results*. For example:

“Lower flow rates allowed more contact time between the elution buffer and the ion exchange resin giving a more concentrated sample…”

could be rewritten as a result first:

“Reducing the flow rate from X to 1 mL/min increased the protein concentration in the eluted fractions (Fig. 3B), likely due to longer resin-buffer contact time.”

Thank you. We have modified sentences in the second paragraph of this section to focus on the result that was observed from protocol changes on page 8 “Removal of impurities and increased concentration through rapid elution of casp-6 in a smaller volume was achieved through elution of casp-6 from the ion-exchange resin in a single-step increase in NaCl concentration instead of a linear gradient (Fig. 3A, 3B)” and “Reducing the flow rate from 5 to 1 mL/min increased the protein concentration in the eluted fractions (Fig. 3B), likely due to longer resin-buffer contact time.”

The improvement is well described qualitatively, but there are **no actual concentration or yield numbers**. Even approximate values (e.g., “final elution concentration increased from ~25 µM to ~60 µM”) would strengthen the claim and allow readers to gauge the benefit.

We thank the reviewer for this point. We have now included concentrations of casp-6 that were observed for the initial and final purification procedures on page 8. “Combining the improved step elution profile (230 mM NaCl), this lower flow rate (1 mL/min) and smaller fraction volumes (0.5 mL) permitted selection of more concentrated samples (Fig. 3B) of ~100 µM instead of 40 µM which was achieved with the traditional elution protocol.”

**pH impacts casp-6 purification and yield:**

**Strengths**

You start by clearly explaining why low pH is desirable for NMR (reduced amide proton exchange), which immediately grounds the pH experiments in their functional relevance.

The decision to stick with pH 8.5 buffers and simplify the workflow (skipping anion exchange) is clearly stated and logically justified.

**Areas for Improvement**

This section mentions “significantly lower yields” but provides no yield values or percentages. Including even approximate numbers (e.g., “yields dropped by ~50%”) would make the impact clear.

This comment was addressed by incorporating specific values on page 9 for the reductions in yield and concentration that were observed when attempting low pH purification of caspase-6. “When Ni-affinity and cation-exchange chromatography were performed at pH 7.5, the yields were <10% of those observed when following the traditional anion-exchange protocol.”

The logic chain from “low pH purification fails” → “high pH works” → “skip anion exchange” is correct, but the transitions could be made smoother by explicitly connecting the findings between each step.

Thank you. We have separated the removal of anion-exchange results into its own section on page 10 and clarified the transitions between these optimization steps. “Following the decreased yield observed with cation-exchange chromatography, we sought to address the necessity of an ion-exchange purification step.”

**Buffer Exchange by desalting column provides the most efficient recovery:**

**Strengths**

The text explains why deuterated buffers are necessary for NMR and why buffer exchange is an unavoidable step.

Provides meaningful parameters (pH, buffer composition, dilution factor, recovery percentages, column type, sample volume, etc.) that allow reproducibility.

Connects changes in pH, glycerol, and DTT to observed precipitation or recovery differences, with mechanistic reasoning.

**Areas for Improvement**

Many sentences run 3–4 lines long, making them harder to follow. Breaking them into shorter, concise statements will improve clarity.

For example, lines 222–229 could be split into two or three sentences to separate findings on *column size* from findings on *sample volume*.

We thank the reviewer for this point. We restructured sentences in this section on pages 11 and 12 to improve clarity and readability. Specifically, splitting long sentences to make more concise statements, and ensuring smooth transitions between ideas. “For example, exchanging a volume of casp-6 smaller than the upper limit for the desalting column resulted in a greater percent recovery of exchanged protein but a larger dilution factor. In contrast, exchanging the maximum volume of casp-6 recommended for the column resulted in a lower dilution factor but not all applied protein was eluted in the recommended elution volume.”

The section moves back and forth between *method comparisons* and *factor influences*. It might read more clearly if you present:

Method comparison results (centrifugal filter vs. desalting column vs. dialysis).

Optimization trials for desalting column (volume, size, pH, glycerol, DTT).

This comment was addressed by ensuring the first paragraph of the corresponding section on pages 10 and 11 discusses the possible exchange methods, while the second paragraph on page 11 discusses optimization of the desalting procedure.

Final recommended conditions. Phrases like "much less casp-6 was lost" could be supported with numerical recovery percentages for precision.

The phrase "significant casp-6 loss to precipitation" would be stronger with quantified loss (e.g., “>40% loss”).

This comment was addressed by the addition of a specific value on page 11 of ~30% observed loss when discussing the absence of glycerol or DTT, or a change in pH. “Decreasing pH (starting buffer Tris pH 8.5) and removal of glycerol or DTT resulted in significant loss of ~30% of casp-6 to precipitation (Fig. 4A, 4B).”

The statement on precipitation due to pH change and isoelectric point crossing is strong but could be made crisper:

*"Loss of protein was likely due to precipitation near its isoelectric point, resulting in instability or increased non-specific binding to Sephadex resin."*

This comment was addressed by altering the corresponding sentence on page 11 to “Loss of protein was likely due to precipitation near its isoelectric point, resulting in instability or increased non-specific binding to Sephadex resin.” to make the point more concisely.

**Tris buffer is optimal for recovery of functional casp-6 and NMR data collection:**

**Strengths**

You connect buffer optimization directly to NMR signal quality and functional enzyme activity, which is exactly the right framing for a methods/results section.

You used both VEID-amc enzymatic assays and ^1H NMR spectra to evaluate buffer conditions, which reinforces confidence in your conclusions. Inclusion of NaCl titrations, pH variations, and multiple buffer additives shows thorough exploration of variables.

**Y**ou explicitly note why certain options (like deuterated BOG) were rejected despite minor improvements, which gives the reader insight into cost–benefit considerations in experimental design.

The observation that FL C163S has sharper amide peaks while D179 CT has better methyl dispersion is valuable for others planning similar experiments.

**Weaknesses / Points to Clarify**

The title “Tris buffer is optimal…” is misleading because your enzyme assay shows *HEPES* gave higher activity, and Tris only stands out for NMR spectral quality. You might want to retitle to something like

*"Balancing enzyme activity and spectral quality: Tris buffer enables optimal casp-6 NMR"*.

We thank the reviewer for this note. This comment was addressed by changing the title to “Tris buffer is optimal to balance casp-6 activity, NMR spectral quality, and recovery during buffer exchange” to emphasize the need for balance between stability and efficient buffer exchange. Additionally, a sentence in the text on page 14 “Taken together with the improved recovery observed in Tris buffer, it was concluded that Tris pH 8.5 would be the preferred buffer for these NMR experiments” was added following discussion of the buffer tests via NMR.

Some figure references (like Fig. 6B) describe "no notable effect" but the visual interpretation might not be obvious to all readers; a short quantitative description (e.g., “<5% variation in peak height”) could help.

This comment was addressed by clarification in the Fig. 6B legend by specifically stating that we were observing peak height and linewidth to assess peak dispersion. “(B) Increasing NaCl concentration resulted in <5% variation in peak height or linewidth in the casp-6 D179 CT ^1^H NMR spectrum.”

**Summary and Conclusions:**

**Strengths**

You connect buffer optimization directly to NMR signal quality and functional enzyme activity, which is exactly the right framing for a methods/results section.

Inclusion of NaCl titrations, pH variations, and multiple buffer additives shows thorough exploration of variables.

You explicitly note why certain options (like deuterated BOG) were rejected despite minor improvements, which gives the reader insight into cost–benefit considerations in experimental design.

The observation that FL C163S has sharper amide peaks while D179 CT has better methyl dispersion is valuable for others planning similar experiments.

**Weaknesses / Points to Clarify**

The title “Tris buffer is optimal…” is misleading because your enzyme assay shows *HEPES* gave higher activity, and Tris only stands out for NMR spectral quality. You might want to retitle to something like *"Balancing enzyme activity and spectral quality: Tris buffer enables optimal casp-6 NMR"*.

This comment appears to have been duplicated and was addressed above.

Some results (e.g., NaCl concentration impact) are discussed twice in slightly different ways, which could be condensed for better flow.

We thank the reviewer for noting this. This comment was addressed on page 14 by rewording the first sentence to clarify that this is what should be expected of ideal NaCl concentrations. This was done by changing “The ideal NaCl concentration balanced sufficient salt for casp-6 to remain folded without decreasing the signal intensity in the ^1^H NMR experiment” to “The ideal NaCl concentration should balance sufficient salt for casp-6 to remain folded without decreasing the signal intensity in the ^1^H NMR experiment.”

Right now, the enzyme assay results and NMR spectral assessments feel somewhat separated. A linking sentence that explicitly states how functional stability trends matched (or didn’t) with NMR signal quality would help the reader.

This comment was addressed by inserting sentences on pages 14 and 15 that tie together the observations from the VEIDase assays and NMR assessment. Specifically, “Additionally, the lack of signal deterioration with increased NaCl concentration in the ^1^H NMR spectra and trends observed for the Tris VEIDase assays (Fig. 5C) is consistent with a previous study that had shown similar concentrations of NaCl were required to stabilize casp-6” and “While the presence of BOG slightly improved signal in the amide region (Fig. 6C), we concluded that the cost associated with exchanging the protein into buffer containing deuterated BOG and the lack of VEIDase improvement (Fig. 5B) outweighed the benefit seen in the ^1^H NMR spectrum.”

Some figure references (like Fig. 6B) describe "no notable effect" but the visual interpretation might not be obvious to all readers; a short quantitative description (e.g., “<5% variation in peak height”) could help.

This comment appears to have been duplicated and was addressed above.

**Materials and Methods:**

**Strengths**:

Procedures are described with sufficient detail to allow replication, including media compositions, induction conditions, and purification steps.

The section follows a natural progression from DNA constructs to expression, purification, activity assays, and finally NMR analysis.

Specifics such as centrifugation speeds, temperatures, buffer compositions, column types, and instrument parameters are well included.

Inclusion of software and statistical methods shows rigor in data handling.

**Suggestions for Improvement:**

Use consistent spacing between numbers and units (e.g., "100 μg/mL" instead of "100 μg / mL"). Spell out abbreviations at first mention if not common, e.g., “OD600 (optical density at 600 nm)”. Standardize temperature formatting: use “20 °C” consistently (you already do well here).

Thank you. We have ensured all units and spacing are uniform throughout, and abbreviations are defined if necessary.

“The cell pellet was then frozen and stored at -80 °C prior to purification” is repeated after different growth conditions — you can state this once after describing all expression protocols.

We changed the end of the second growth protocol on page 21 to reference the details outlined in the first growth protocol, resulting in the sentence “Overexpression, cell pellet harvesting, and storage was then carried out as described for minimal media cultures.”

In line 426, you wrote “100 μg/mL ampicillin for all caspase-6 constructs.” Then in line 432, “100 mg/L ampicillin.” Though equivalent, use one unit consistently (preferably μg/mL in microbiology context).

This comment was addressed by changing all references to ampicillin to 100 μg/mL.

When listing buffer components (lines 430-434), consider using a table or bullet list in supplementary materials for clarity if journal allows.

This comment was addressed by reference on page 20 to the existing table “S1 Table” which contains these media components.

In line 466: the use of “linear gradient to 15% buffer B” — specify if this means 15% v/v or something else for precision.

This comment was addressed by inserting the detail “v/v” on page 22.

The NMR methods are detailed, but consider briefly explaining acronyms like HMQC and SOFAST for broader accessibility.

This comment was addressed by defining these abbreviations in the “NMR Data Collection” methods section on page 24.

Mention the rationale for choosing specific constructs for different experiments if relevant (e.g., why 140 μM for FL vs 30-70 μM for D179 CT).

This comment was addressed on page 24 by the addition of a sentence describing the reasoning for protein concentrations “The protein concentration in NMR was dependent on two factors: the yield following purification and the minimal concentration required to obtain sufficient NMR signal.”

Clarify if temperature variations between 20 °C and 35 °C were for specific purposes or instrument constraints.

This comment was addressed by the addition of a sentence on page 24 describing the choice for higher temperature for the SOFAST spectrum “Higher temperature was used for the SOFAST spectrum to increase signal-to-noise.”

You mention multiple R packages and correction methods, which is excellent. Consider briefly mentioning the rationale for choosing Benjamini-Hochberg correction over others.

This comment was addressed by a brief explanation on page 23 stating that Benjamini-Hochberg correction was deemed more suitable for the exploratory nature of this research “Where applicable, multiple comparisons were corrected using the Benjamini-Hochberg (BH) method as this was found suitable for analyzing exploratory trends.”

If the Methods section is too long for the main text, some of the detailed buffer recipes and NMR parameters might be moved to Supplementary Materials with a brief overview retained here.

Thank you for this suggestion. We also considered this approach. Ultimately, given that *PLOS ONE has* no word limits, we concluded it was easier for the readers if the Methods section was included in the main text. If the editorial team suggests this moving it, we will certainly comply.

**Summary:**

Your **Materials and Methods** section is very solid and well-written. Minor tweaks for consistency, clarity, and readability will polish it further and improve accessibility for diverse readers. Including rationale for some parameter choices would strengthen the scientific narrative.

Thank you so much for your time in reviewing our manuscript. We have never had a more thoughtful analysis of any paper. We very much appreciate your dedication, your insights and your effort!
